# Abundance‐mediated species interactions

**DOI:** 10.1002/ecy.4468

**Published:** 2024-12-05

**Authors:** Joshua P. Twining, Ben C. Augustine, J. Andrew Royle, Angela K. Fuller

**Affiliations:** ^1^ New York Cooperative Fish and Wildlife Research Unit, Department of Natural Resources and the Environment Cornell University Fernow Hall Ithaca New York USA; ^2^ Department of Fisheries, Wildlife, and Conservation Science Oregon State University Nash Hall Corvallis Oregon USA; ^3^ U.S. Geological Survey Northern Rocky Mountain Science Center Bozeman Montana USA; ^4^ Eastern Ecological Science Center U.S. Geological Survey Laurel Maryland USA; ^5^ U.S. Geological Survey, New York Cooperative Fish and Wildlife Research Unit, Department of Natural Resources and the Environment Cornell University Ithaca New York USA

**Keywords:** abundance, density dependent, hierarchical models, interaction networks, occupancy, Royle–Nichols, species interactions, wildlife monitoring

## Abstract

Species interactions shape biodiversity patterns, community assemblage, and the dynamics of wildlife populations. Ecological theory posits that the strength of interspecific interactions is fundamentally underpinned by the population sizes of the involved species. Nonetheless, prevalent approaches for modeling species interactions predominantly center around occupancy states. Here, we use simulations to illuminate the inadequacies of modeling species interactions solely as a function of occupancy, as is common practice in ecology. We demonstrate erroneous inference into species interactions due to error in parameter estimates when considering species occupancy alone. To address this critical issue, we propose, develop, and demonstrate an abundance‐mediated interaction framework designed explicitly for modeling species interactions involving two or more species from detection/non‐detection data. We present Markov chain Monte Carlo (MCMC) samplers tailored for diverse ecological scenarios, including intraguild predation, disease‐ or predator‐mediated competition, and trophic cascades. Illustrating the practical implications of our approach, we compare inference from modeling the interactions in a three‐species network involving coyotes (*Canis latrans*), fishers (*Pekania pennanti*), and American marten (*Martes americana*) in North America as a function of occupancy states and as a function of abundance. When modeling interactions as a function of abundance rather than occupancy, we uncover previously unidentified interactions. Our study emphasizes that accounting for abundance‐mediated interactions rather than simple co‐occurrence patterns can fundamentally alter our comprehension of system dynamics. Through an empirical case study and comprehensive simulations, we demonstrate the importance of accounting for abundance when modeling species interactions, and we present a statistical framework equipped with MCMC samplers to achieve this paradigm shift in ecological research.

## INTRODUCTION

Ecological community assemblage emerges from interactions between competition, predation, and disease, and one of the greatest contemporary issues facing ecologists is to determine how and to what extent interspecific interactions drive species coexistence, community structure, and biodiversity patterns (Morin, 2011). Using existing modeling frameworks, predation, herbivory, competition, and parasitism have all been identified as having strong influences on system processes at both local and landscape‐scales (Bonsall & Holt, 2003; Hatcher et al., 2006; Holt, 1977; Slade et al., 2023; Tompkins et al., 2003). The challenges of scaling up such interactions to spatial scales more relevant to species and their conservation are evident when we consider that interactions vary in their strength and direction, dependent on local habitat (Tompkins et al., 2003; Twining, Sutherland, et al., 2022), community composition (e.g., presence of numerically dominant prey; Slade et al., 2022, a shared predator; Twining, Lawton, et al., 2022 or a parasite/pathogen; Hatcher et al., 2006), and the population sizes of species of interest (Slade et al., 2023).

In the past, accounting for species interactions has primarily been achieved by (1) simply modeling unadjusted counts of observations/detections, or indices of relative abundance, whereby imperfect detection is ignored (e.g., Allen et al., 2019; Latimer et al., 2009; Pollock et al., 2014), or (2) through hierarchical‐based frameworks whereby imperfect detection is explicitly accounted for, including (1) joint species distribution/community models (e.g., Doser et al., 2023; Tobler et al., 2019), (2) two‐species occupancy models (e.g., Richmond et al., 2010; Waddle et al., 2010), and (3) multivariate Bernoulli (MVB) multispecies occupancy models (e.g., Rota et al., 2016). All the hierarchical models above leverage detection/non‐detection data of species collected through repeated surveys. This sampling approach enables the user to account for the observation processes responsible for imperfect detection (Richmond et al., 2010; Rota et al., 2016; Tobler et al., 2019). The recognition of the importance of accounting for imperfect detection and growing use of hierarchical modeling frameworks to achieve explicit incorporation of the observation process has increased the robustness of inferences into estimates of species distributions, abundance, and interactions (MacKenzie et al., 2002, 2006; Rota et al., 2016; Royle, 2004; Royle & Nichols, 2003).

One of the most widely applied hierarchical co‐occurrence models is the Rota et al. (2016) multispecies occupancy model. This model facilitates the estimation of species probability of occupancy conditional on the presence or absence of another species by assuming the latent occupancy state is a MVB random variable. Logic suggests that in an idealized occupancy survey, the Rota et al. model can be highly effective when the interactions between the species of interest are very strong, and abundances do not vary considerably. Critically, the model will likely provide useful inference into interactions where the presence of one species alone is sufficient to predictably result in the exclusion or occurrence of another. For example, in Japan, invasive raccoon (*Procyon lotor*) occurrence was suppressed, conditional on the presence of the native tanuki (raccoon dog, *Nyctereutes procyonoides viverrinus*; Kass et al., 2020). While cases where a species presence may influence the colonization or extinction probabilities of another may be relatively common, cases where interactions between species are sufficiently strong that the presence of a species alone is enough to predictably result in the exclusion of another, are by their nature, rare in natural systems (Twining, Lawton, et al., 2022).

Interactions can involve direct (e.g., predation, competition, disease) and/or indirect (e.g., apparent competition, predator/disease mediated competition) pathways, but these are commonly countered by mechanisms for coexistence (e.g., temporal or spatial avoidance) and critically, are mediated by species abundance. For example, lions (*Panthera leo*) suppress African wild dogs (*Lycaon pictus*) through intraguild killing and kleptoparasitism, but wild dogs use spatial heterogeneity and fine‐scale habitat selection to avoid dominant lions, facilitating the two species coexistence (Davies et al., 2021). In a scenario such as this one, despite a wealth of evidence demonstrating negative interactions between the species in question (Goodheart et al., 2022; Mills & Biggs, 1993; Mills & Gorman, 1977; Swanson et al., 2014), we posit that considering only the occupancy states of the two species could erroneously result in estimation of a positive or independent interaction term as the species co‐occur widely. The former could be misinterpreted as conditional occurrence of lions benefitting wild dogs, and the latter of the species not interacting. In an alternate example, in a system with variable species abundances and density‐dependent interactions, for example, the case of an introduced predator preying upon a native prey species, such as seen in the interactions between invasive American mink (*Neovison vison*) and native European water voles (*Arvicola amphibius*) in Great Britain (Oliver et al., 2009), modeling these density‐dependent interactions as a function of occupancy states would result in overestimation of interaction strength. Here, we see that different ecological systems provide variable possibilities for how modeling interactions as a function of occupancy states may result in erroneous parameter estimates. Understanding species interactions on spatial scales relevant to the management and conservation of wildlife populations is of critical importance, yet co‐occurrence‐based frameworks are frequently applied indiscriminately, and without due caution, potentially leading to erroneous inference into systems of interest, and thus the potential for misguided management.

Frequency, and thus strength of species interactions, is highly dependent on local densities of the species of interest; critically, interactions between species are intrinsically mediated by population size of the species of interest (Kawatsu & Kondoh, 2018; Lotka, 1925). Recognition of the critical importance of abundance in mediating the outcomes of species interactions dates back to initial attempts to understand interspecific interactions and community dynamics using classic Lotka–Volterra equation–based modeling (e.g., Anderson & May, 1981; Gauze, 1934; Holt, 1977; Lotka, 1925; Murdoch, 1969). Novel adaptations and applications of these classic approaches continue to provide novel insights into species interactions and ecological systems today (e.g., Slade et al., 2023; Tanner et al., 2019). The common focus on occupancy in the modeling of species interactions from empirical data stems from the ease of data collection, as opposed to the relevance of the state variable. A biologically appropriate interaction model would model species abundance, not species occupancy. The spatial capture–recapture (SCR) family of models (Royle et al., 2014) would be the ideal framework for addressing abundance‐mediated interactions (e.g. Gaya & Chandler, 2024); however, these models are dependent on data pertaining to identity of individuals detected, which can be prohibitively costly to collect (e.g., Twining, MacFarlane et al., 2022 ), and often logistically implausible to conduct on sufficiently large scales, even for single species. This problem is compounded in many urgent conservation situations, where the target species is rare, elusive, or otherwise difficult to detect. In recent years, a range of models for estimating species from unmarked counts while accounting for imperfect detection have been developed. For example, latent‐ID SCR models use underlying spatial correlations of counts to probabilistically assign individual identity to samples within a spatial capture‐recapture framework (e.g., Augustine et al., 2020; Chandler & Royle, 2013), while N‐mixture and Royle–Nichols (R–N) models rely on assumptions of no individual heterogeneity in detection to model unmarked independent and spatially replicated counts or detection/non‐detection data to provide inference into both abundance and individual detection probability (Royle, 2004; Royle & Nichols, 2003). Nevertheless, the efficacy of a model framework which explicitly models the occupancy or abundance of one species as a function of the abundance of one or more other species from detection/non‐detection data and allows the interaction term to vary as a function of the environment remains undemonstrated.

Habitat and environmental variation can mediate interactions (Kass et al., 2020; Rota et al., 2016). For example, past research has shown how in forested landscapes with low levels of non‐native timber plantation cover the presence of a native predator; the pine marten (*Martes martes*) increases the occurrence of its native prey, the threatened red squirrel (*Sciurus vulgaris*) through the control of an invasive competitor; the grey squirrel (*Sciurus carolinensis*) through predator‐mediated competition. However, the strength and direction of this interaction is reversed when simple non‐native timber plantations dominate the landscape; the presence of pine marten in these areas reduces the occurrence of the red squirrel (Twining, Sutherland, et al., 2022). This example highlights the dynamic nature of interactions, and the importance for any biologically feasible model for inference into species interactions to be able to account for spatial and temporal heterogeneity in interactions.

We use simulation to test and demonstrate the impacts on inference resultant from modeling interactions as being mediated by occupancy versus abundance. We then propose an extension of the Waddle et al. (2010) model to incorporate abundance‐mediated interactions between two or more species, and additionally allow each component of the model (abundance, occupancy, and interaction terms between the species) to vary both spatially, and temporally. Our model framework uses detection/non‐detection data and leverages well‐established R–N (Royle & Nichols, 2003) and occupancy models (MacKenzie et al., 2002). We use multiple simulation studies to demonstrate capabilities of the model framework under different sampling regimes and ecological scenarios (variable site numbers and sampling occasions, both two‐ and three‐species interactions, constant or variable interaction terms), and then, as an empirical example, we model the interactions between coyotes (*Canis latrans*), fishers (*Pekania pennanti*), and the American marten (*Martes americana*) on a landscape‐scale across northeastern New York State.

## METHODS

### Data description

The required sampling protocol and assumptions for this abundance‐mediated interaction model are the same as for a closed system single‐species occupancy model (MacKenzie et al., 2002) and the R–N model (Royle & Nichols, 2003). The model requires *a priori* specification of directionality of interactions with identification of dominant and subordinate species (with addition of intermediate species possible for interactions involving >2 species). For sampling, a number of independent sites are selected from an area of interest, and each site *i* is repeatedly sampled *j* times. Every occasion *j* can be formed of *k* subsamples (these could be intervals through time or space—e.g., subsampling *k* nights in weekly occasion *j* or *k* segments of a transect). $y_{i,j,k}^{S}$ and $y_{i,j,k}^{D}$ are the detection histories of the subordinate species, *S*, and the dominant species, *D*, for site *i* = 1, …, *I*, occasion *j* = 1, …, *J*, and subsample *k* = 1, …, *K*. Detection/non‐detection data are partial observations of the underlying abundance state of the dominant species which we assume can be modeled as a series of binomial trials of site by occasion‐level detection probability *p* ^*D*^. Detection/non‐detection data of the subordinate species can be modeled as either partial observations of the species underlying abundance state, akin to the dominant species, or considered partial observations of the subordinate species occupancy state which we assume can be modeled as a Bernoulli random variable.

The key assumptions of the model are (1) population closure—the population is closed during sampling, with no deaths, births, immigration, or emigration, all within‐site variation in detection/non‐detection data or counts is due to variation across sites in individual detection probability and abundance; (2) no false positives—no mistaken identification of species, and no double counting of individuals; (3) spatial independence of occupancy or abundance states—no individual can be detected at more than one detector; (4) no unmodelled heterogeneity—there is no unaccounted‐for heterogeneity in either the state or the observation model; and (5) no individual heterogeneity in *p*, while detection probability may vary as a function of covariates, once this has been accounted for, detection probability is assumed to be constant across individuals.

### Abundance‐mediated interaction model

We extend the interaction formula from Waddle et al. (2010) which used the latent presence/absence (*z* state) of a species as a covariate in the state model of another species. We adapt this formulation to an abundance‐mediated interaction model, where the dominant species covariate is no longer a binary *z* state but *N*, the abundance of the dominant species (which can take the value of any non‐negative integer). The abundance submodel is a R–N model (Royle & Nichols, 2003), modeling count data formed from summarized detection/non‐detection using a binomial formulation for the observation model. This observation model exploits the link between heterogeneity in abundance and heterogeneity in site‐level detection probability to estimate the underlying distribution of abundances and account for site level heterogeneity in detection.

In the state model for the dominant species, the number of individuals at site *i*, $N_{i}^{D}$, is modeled as being drawn from a Poisson distribution of parameter $\lambda_{i}$, where $\log\left(\lambda_{i}\right)$ is modeled as a function of site covariates:

$$N_{i}^{D}\sim \text{Poisson}\left(\lambda_{i}\right)$$

We consider a situation whereby observations are independent. In this R–N observation model, we assume that the site by occasion by subsample probability of detection is a function individual detection probability and the number of individuals at a site:

$$p_{\mathrm{ij}}^{D}=1-{\left(1-r_{\mathrm{ij}}^{D}\right)}^{N_{i}^{D}}$$

where $p_{\mathrm{ij}}^{D}$ is the probability one or more individuals are detected at site *i* on occasion *j* in subsample *k*, $r_{\mathrm{ij}}^{D}$ is individual level detection probability at site *i* on occasion *j*, and $N_{i}^{D}$ is the number of individuals at site *i*, regarded as a latent variable here. Individual detection probability $r_{\mathrm{ij}}^{D}$ is modeled as a function of covariates, and thus while constant for the site and occasion during a given sample, $r_{\mathrm{ij}}^{D}$ can vary across time and space. The observed count data ($y_{\mathrm{ij}}^{D}$) at site *i* on occasion *j* are modeled as a random draw from *K* binomial trials with site by occasion by subsample level detection probability ($p_{\mathrm{ij}}^{D}$):

$$y_{\mathrm{ij}}^{D}\sim \text{Binomial}\left(p_{\mathrm{ij}}^{D},K\right)$$

This results in a detection model that is naturally suited to many mainstream sampling methods. The value of *K*, the number of intervals within an occasion, is dependent on study design, the sampling methodology, and/or the length of the occasion being used. For example, *K* could be set to 1 for standard detection/non‐detection data or increased to include *K* number of sampling intervals within an occasion for count data formed of repeat detection/non‐detection events; for example, if conducting repeated line transects, the transect could be divided into *K*‐segments.

The model for the subordinate species could be formulated as a R–N model to estimate abundance, $N_{i}^{S}$, as seen above for the dominant species, or it can be formulated to estimate occupancy, $z_{i}^{S}$, following the standard single species occupancy model of MacKenzie et al. (2002), where the state model for the latent *z* state of the subordinate species at site *i* is modeled as a Bernoulli random variable:

$$z_{i}^{S}\sim \text{Bernoulli}\left(\varphi_{i}^{S}\right)$$

Here, we assume that the observed detection/non‐detection data for the subordinate species ($y_{\mathrm{ij}}^{s}$) is a function of the detection probability of the subordinate species ($p_{\mathrm{ij}}^{S}$), conditional on its presence:

$$y_{\mathrm{ij}}^{S}\sim \text{Bernoulli}\left(p_{\mathrm{ij}}^{S}\;\times \;z_{i}^{S}\right)$$

We model variation in both the observation and state model parameters using covariates on the appropriate link scale, for example, in the state model for the dominant species:

$$\log\left(\lambda_{i}^{D}\right)=\beta_{0}+\beta_{1}\times {\text{covariate}}_{i}^{1}+\beta_{2}\times {\text{covariate}}_{i}^{2}$$

In the subordinate species, we extend the Waddle et al. formulation of species interactions where the presence/abundance or detection of the subordinate species is assumed to be dependent upon the latent *z* state of a dominant species. In our extension, we assume that the presence/abundance and detection of the subordinate species can be dependent on the abundance of the dominant species $\left(N_{i}^{D}\right)$, and similar to the Waddle et al. model, this interaction can vary as a function of environmental covariate *x*  ($N_{i}^{D}\times {\text{covariate}\;}_{i}^{x}$), for example, for an occupancy state model:

$$\text{logit}\left(\psi_{i}^{S}\right)=\beta_{0}+\beta_{1}\times {\text{covariate}}_{i}^{1}+\gamma_{0}\times N_{i}^{D}+\gamma_{1}\times N_{i}^{D}\times {\text{covariate}\;}_{i}^{2}$$

**FIGURE 1:**
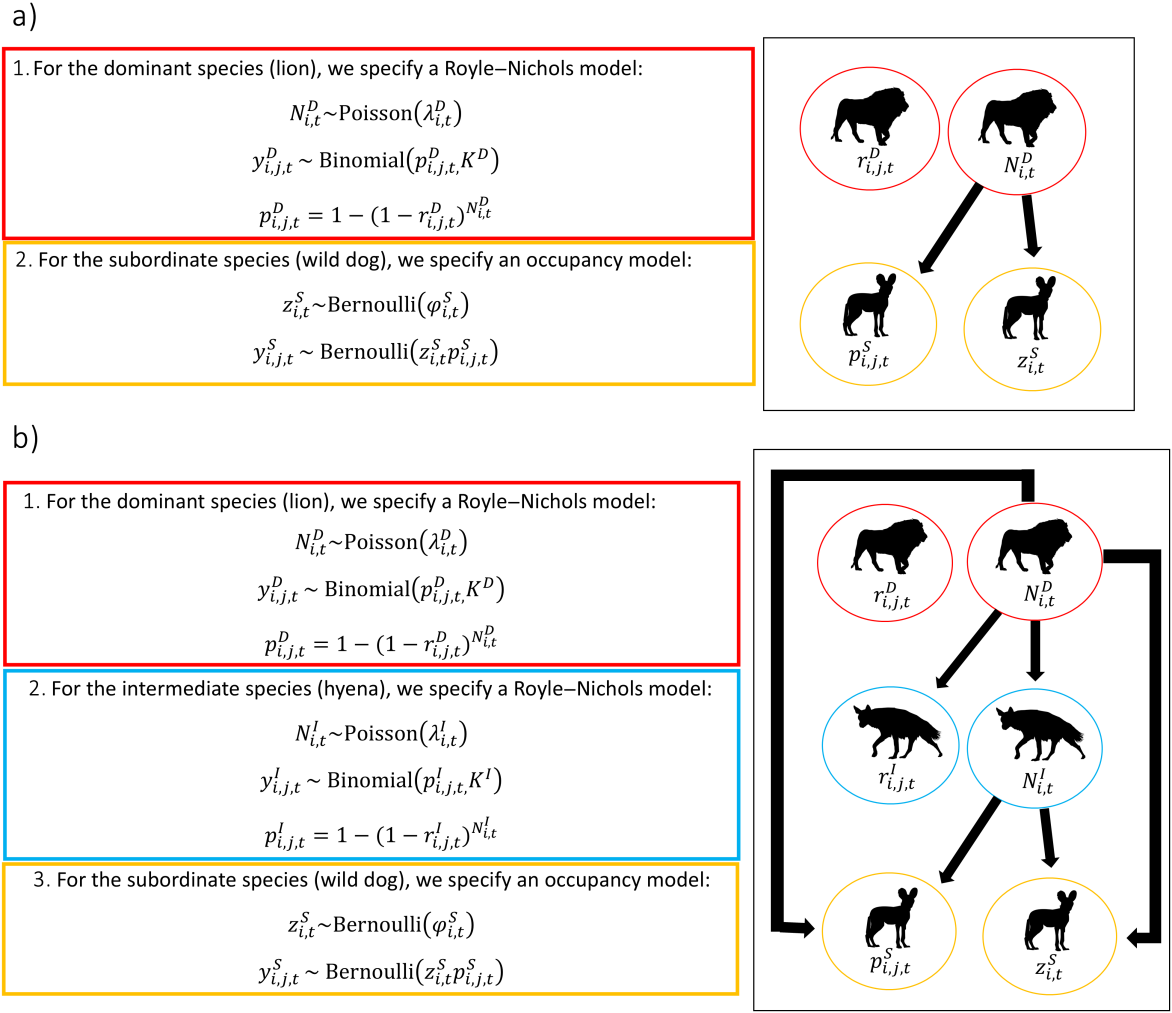
A conceptual diagram of (a) a hypothetical two‐species abundance‐mediated interaction model between lions (*Panthera leo*) and wild dogs (*Lycaon pictus*), and (b) a three‐species abundance‐mediated interaction model between lions, hyena (*Parahyaena brunnea*), and wild dogs, with direction of interactions specified based on *a priori* hypotheses. Illustrations created by Margot Michaud and Lisa Nicvert available under CC0 1.0 Universal Public Domain Dedication licenses.

Different hypotheses regarding the importance of environmental variables and species interactions can be examined by specifying different formulations of the above regression models. For example, this could be achieved through comparison of models which assumes a species occurrence is only driven by environmental covariates ($m_{1}=\beta_{0}+\beta_{1}\times {\text{covariate}}_{i}^{1}+\beta_{2}\times {\text{covariate}\;}_{i}^{2}$) with another that assumes it is a combination of both environmental covariates and the abundance of another species ($m_{2}=\beta_{0}+\beta_{1}\times {\text{covariate}}_{i}^{1}+\gamma_{0}\times N_{i}^{D}$). An advantage of this model formulation in addition to incorporating information on species abundance is that the interaction terms can be modified as a function of covariates (as seen above). This model requires *a priori* specification of the directionality of interactions, which thus necessitates clear *a priori* hypotheses regarding the system of interest (a standard prerequisite for ecological research). This model could naturally be extended to three or more species by increasing the number of integrated submodels, although directionality must be specified (see Figure 1a for two species example, and Figure 1b for three‐species example).

**FIGURE 2:**
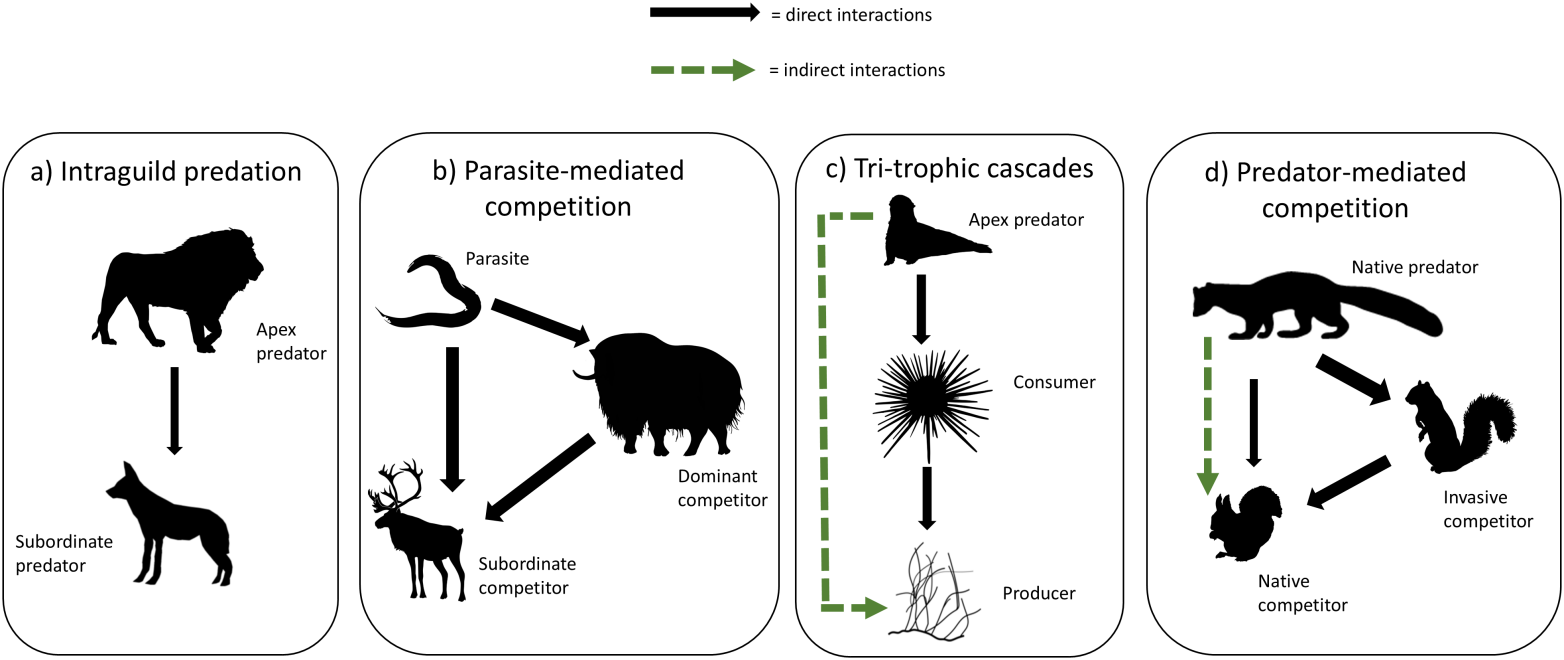
Diagram of different ecological contexts that an abundance‐mediated interaction modeling framework could be applicable including (a) intraguild predation, (b) parasite‐mediated competition, (c) tri‐trophic cascades, and (d) predator‐mediated competition. Black solid lines show direct effects, while green dashed lines show indirect effects. Line thickness indicates strength of the interaction. See Figure 1 and Appendix S1: Figures S1–S3 for conceptual diagrams and corresponding code for ecological contexts (b), (c), and (d), which vary in their structure, being abundance–occupancy–occupancy, abundance–abundance–abundance, and occupancy–abundance–abundance models, respectively. Illustrations created by Margot Michaud, Jake Warner, Laura Barbero‐Palacios, Holly English, Guillaume Dera, Harold N. Eyster, Ferran Sayol, and Andy Wilson available under CC0 1.0 Universal Public Domain Dedication licenses; illustrations were not modified.

This framework is highly flexible and could be adapted to a variety of ecological community formations of conservation interest. Examples include competitively linked prey species with shared predators, tri‐trophic cascades, and pathogen/parasite mediated competition (see Figure 2). The framework is naturally suited to hypothesis testing, as it requires *a priori* specification of directionality of interactions. Additionally, the state variable (*z* or *N*) being estimated in each species submodel can be changed as appropriate to the context of interest. See Appendix S1 for various ecological scenarios and model configurations (abundance–occupancy–occupancy, abundance–abundance–abundance, occupancy–abundance–abundance) with motivating case studies, model code, and data simulators. We develop the base model and variants and conduct all modeling using C++ via the NIMBLE package version 0.13.1 (de Valpine et al., 2022) in R.

### Simulation study I: A comparison of modeling species interactions as occupancy versus abundance‐mediated

We conducted a number of simulation studies to assess the ability of the abundance‐mediated interaction model to identify and estimate interaction parameters accurately in different situations. We simulated data directly from the abundance‐mediated interaction model under different scenarios. In the first study, we examine the impact of modeling abundance‐mediated species interactions as a function of occupancy versus as a function of abundance, and how the abundance of the species of interest mediates the accuracy of parameter estimates when modeling abundance‐mediated interactions as a function of occupancy compared to abundance. We simulated two species with constant detection probabilities (*r* ^*D*^ = 0.3, *p* ^*S*^ = 0.5), and set a constant mean occupancy state for the subordinate species (ψ^*s*^ = 0.75). We set constant interaction terms as a function of abundance between the two species (γ_0_ = −1, γ_1_ = 0), which we assume to be representative of real‐world interactions. The number of sites sampled (*I* = 600) and occasions per site (*J* = 4) were kept constant. This resulted in three simulation scenarios of varying dominant species abundances. For each of the three unique combinations of parameters in the simulation study, we simulated 250 datasets from the model and fit the model in two ways: (1) with interactions modeled as a function of occupancy and (2) with interactions modeled as a function of abundance for a total of six evaluations. These datasets included detection/non‐detection data summarized as count data for the dominant species, and detection/non‐detection data for the subordinate species. We considered a single closed sampling period. We fit the same specification of the abundance‐mediated interaction model to each dataset, only varying whether we modeled the interactions as a function of *z* ^*D*^ (occupancy state) or *N* ^*D*^ (abundance state) of the dominant species for each of the mean population abundance values. All models ran three chains for 50,000 iterations with effective thinning of 45,000, resulting in 5,000 posterior samples. We used vague priors for all parameters (see Appendix S1: Table S1). We recorded posterior modes and credible intervals for all parameters. We calculated mean relative bias and coverage of all parameters using only models that had converged (using Gelman–Rubin diagnostics, $\hat{R}>1.1$; Gelman & Rubin, 1992). For non‐zero parameter values, relative bias was calculated as:

$${\mathrm{RB}}_{x}=\frac{{\hat{ϴ}}_{x}-{ϴ}_{x}}{{ϴ}_{x}}$$

where RB is relative bias of parameter *x*, $\hat{ϴ}$ is the model estimate for parameter *x*, and ϴ is the true value of parameter *x*. For parameter values of zero, absolute bias in parameter estimates was calculated as:

$${\mathrm{AB}}_{x}={\hat{ϴ}}_{x}-{ϴ}_{x}$$

We define negligible bias or unbiased parameter estimates as those with relative bias (or absolute bias for parameters with true values of zero) of less than or equal to ±0.05; anything over this threshold, we consider to be biased. Where relevant we report whether errors were type S (sign) errors (where the direction of the effect estimated is incorrect) or type M (magnitude) errors where an effect is overestimated or underestimated (Gelman & Carlin, 2014). We estimated coverage for each parameter by dividing the number of simulated data sets for which the 95% credible interval covered the true parameter value by the number of simulations in a scenario. We report the proportion of simulation repetitions where the true value of parameter *x* was within the 95% credible intervals for the estimate of parameter *x* using 0.9 as our threshold for acceptable coverage.

### Simulation study II: Two‐species simulation with spatially variable complexity and variable sampling effort

We simulated two species with constant detection probabilities (*r* ^*D*^ = 0.5, *p* ^*S*^ = 0.5), a mean population size of λ^*D*^ = 1 for the dominant species, and a mean occupancy of ψ^*s*^ = 0.5 for subordinate species. We varied the number of sampling sites to examine the number of spatial replicates (*I* = 300, 600, and 1000 sites) required to obtain unbiased parameter estimates. We also varied the number of sampling occasions per site (*J* = 4, 10). Then, we specified the model with increasing levels of complexity starting with a simple constant interaction term on the state model (γ_0_ = −1, γ_1_ = 0), then adding an interaction term that varied as a function of an environmental covariate (γ_0_ = −1, γ_1_ = 1). For each unique combination of parameters (12 simulation scenarios from the combination of three spatial replicate values, two sampling occasion values, and two complexity variants) in the simulation study, we simulated 250 datasets from the model. We fit the appropriate specification of the abundance‐mediated interaction model to each dataset. For all models, we ran three chains for 50,000 iterations with effective thinning of 45,000, resulting in 5,000 posterior samples. We used vague priors for all parameters (see Appendix S1: Table S1). We calculated relative bias and coverage as conducted in simulation study I.

### Simulation study III: Three‐species simulation with constant interaction terms and variable detection probabilities

We conducted a third simulation study to assess the ability of abundance‐mediated interaction models to estimate parameters accurately in a three‐species scenario with variable detection probabilities. We simulated data directly from the abundance‐mediated interaction model. We simulated populations with constant mean population sizes λ^*D*^ = 0.5 for the dominant species, λ^*I*^ = 0.5 for the subordinate species, and constant mean occupancy, ψ^*s*^ = 0.5, for the subordinate species, at a constant number of spatial replicates (*I* = 600). Each site sampled had four sampling occasions. We specified the model to have a constant direct negative interaction term between the dominant and intermediate species (γ_0_^*D*‐*I*^ = −1), a constant direct negative interaction between the intermediate and subordinate species (γ_0_^*I‐S*^ = −1), and a constant direct positive interaction between the dominant and subordinate species (γ_0_^*D‐S*^ = 1). To test parameter estimate sensitivity to detection probability and the hypothesis that estimates of interaction parameters are sensitive to detection probability of species, we consider both high and low detection probabilities for both the dominant (*r* ^*D*^ = 0.05–0.5), and subordinate species (*p* ^*S*^ = 0.25–0.5), while keeping the intermediate species individual detection probability constant (*r* ^*I*^ = 0.5). For each unique combination of parameters (four), we simulated 250 datasets from the model. We fit the appropriate specification of the abundance‐mediated interaction model to each dataset. All models ran three chains for 50,000 iterations with a thinning rate of 2, and a burn in of 20,000, resulting in 5,000 posterior samples. We used vague priors for all parameters. We recorded posterior modes and credible intervals for all parameters. We calculated relative bias and coverage of all parameters using only models that had converged (using Gelman–Rubin diagnostics, $\hat{R}>1.1$).

### Case study: Intraguild interactions between coyotes, fishers, and American martens

In the case study using empirical data, we examine the intraguild interactions between three carnivores: a top mesopredator in the system, the coyote, an intermediate mesopredator, the fisher, and a small carnivore, the American marten. There is a long history of examining intraguild interactions between fisher and marten through harvest data (e.g., Hardy, 1907; Krohn et al., 1997). Recent harvest‐based evidence was used to infer negative interactions between all three species, with fishers being limited through intraguild killing by coyotes, and martens being limited by both fisher and coyotes (Jensen & Humpries, 2019). Despite this, the three species co‐occur over much of the marten's limited range in New York State and recent analysis using Rota et al. (2016) co‐occurrence models produced results inconsistent with previous hypotheses. The co‐occurrence analysis found fisher occupancy was higher conditional on coyote presence, and marten occurred independently from both other species (Twining, Brazeal, et al., 2024). Nonetheless, as explored in Simulation study I, a focus on occupancy states (and ignoring the abundance of species) to infer interactions may limit inference. As an example, we fit an abundance‐mediated interaction model to the landscape scale camera trapping dataset on these species previously analyzed using co‐occurrence models in Twining, Brazeal, et al. (2024). Detection frequency data on coyotes, fisher, and American marten were obtained from camera traps deployed to monitor occurrence of the target species in northeastern New York State. The dataset was collected by the New York State Department of Environmental Conservation and is available online (see Twining, Jensen, et al., 2024); field data collection details are described in full in Twining, Brazeal, et al. (2024). Briefly, sampling was conducted from January to March 2016–2018 throughout the Adirondack and Tug Hill regions of northern New York State. A standardized methodology was used to survey 195 15‐km^2^ sample units across the region of interest. The spatial scale of sample units (15 km^2^) minimized violations of spatial independence assumptions given reported home ranges for the three species (see Twining, Brazeal, et al., 2024). At each site, a camera trap was deployed randomly within the 15‐km^2^ grid. Camera traps were secured to trees approximately 1.0–1.5 m above ground. A bait station was placed on a tree opposite the camera trap. Cameras were deployed for 3 weeks (21 days) at each location after which cameras were retrieved. As in the previous analysis (Twining, Brazeal, et al., 2024), we used a weekly occasion length. We allowed a single detection each 24‐h period over each week and summed the days with detections into counts composed of seven daily subsamples (*K*). The same sample units were sampled in each of the three sampling years (except for 13 sites that were not sampled in 2017). Thus, to account for temporal heterogeneity and dependence across the three sampling years, we indexed the data over years and estimated intercepts for each year on all three species state and observation models (as we did not have sufficient primary sampling periods to support yearly random effects).

We fit and interpreted a global model, full details of which can be found in Appendix S1: Case study—three species abundance‐mediated interactions between coyotes–fishers–marten in northeastern North America. For this example, we were primarily interested in whether the estimated coyote abundance impacted fisher abundance (γ_0_^*C‐F*^), and whether coyote or fisher abundance impacted marten abundance (γ_0_^*C‐M*^; γ_0_^*F‐M*^). We fit three constant interaction terms between the species (one in the fisher state model and two in the marten state model). We ran three chains for 200,000 iterations, with a thinning rate of 8, and a burn in of 20,000, resulting in 5,000 posterior samples. We used vague priors for all parameters. Models were considered to have converged only if visual inspection of chains showed good mixing for all parameters and upper CIs for $\hat{r}$ values from Gelman–Rubin diagnostics were <1.1 (Gelman et al., 2013). We used posterior predictive checks with a Pearson chi‐square fit statistic for each species submodel to ensure there was no evidence of lack of fit (Gelman et al., 1996). We provide evidence of variable effects by calculating 95% credible intervals for each parameter and identifying those which do not overlap 0.

## RESULTS

### Simulation study I: A comparison of modeling species interactions as occupancy versus abundance‐mediated

**FIGURE 3:**
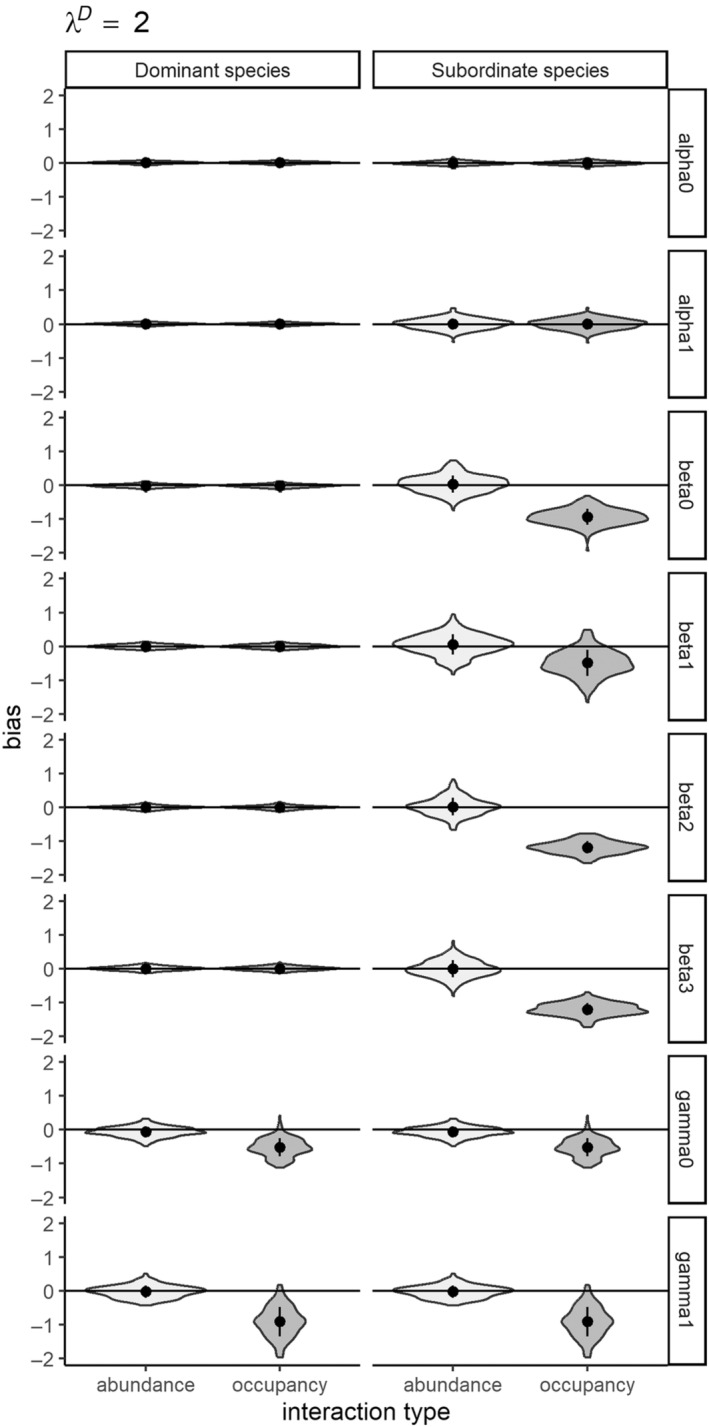
Violin plots showing relative error of parameter estimates from simulation study I (a comparison of modeling species interactions as occupancy‐ vs. abundance‐mediated) which compares modeling interactions as a function of abundance to modeling interactions as function of occupancy states for a highly abundant dominant species (λ^*D*^ = 2).

**FIGURE 4:**
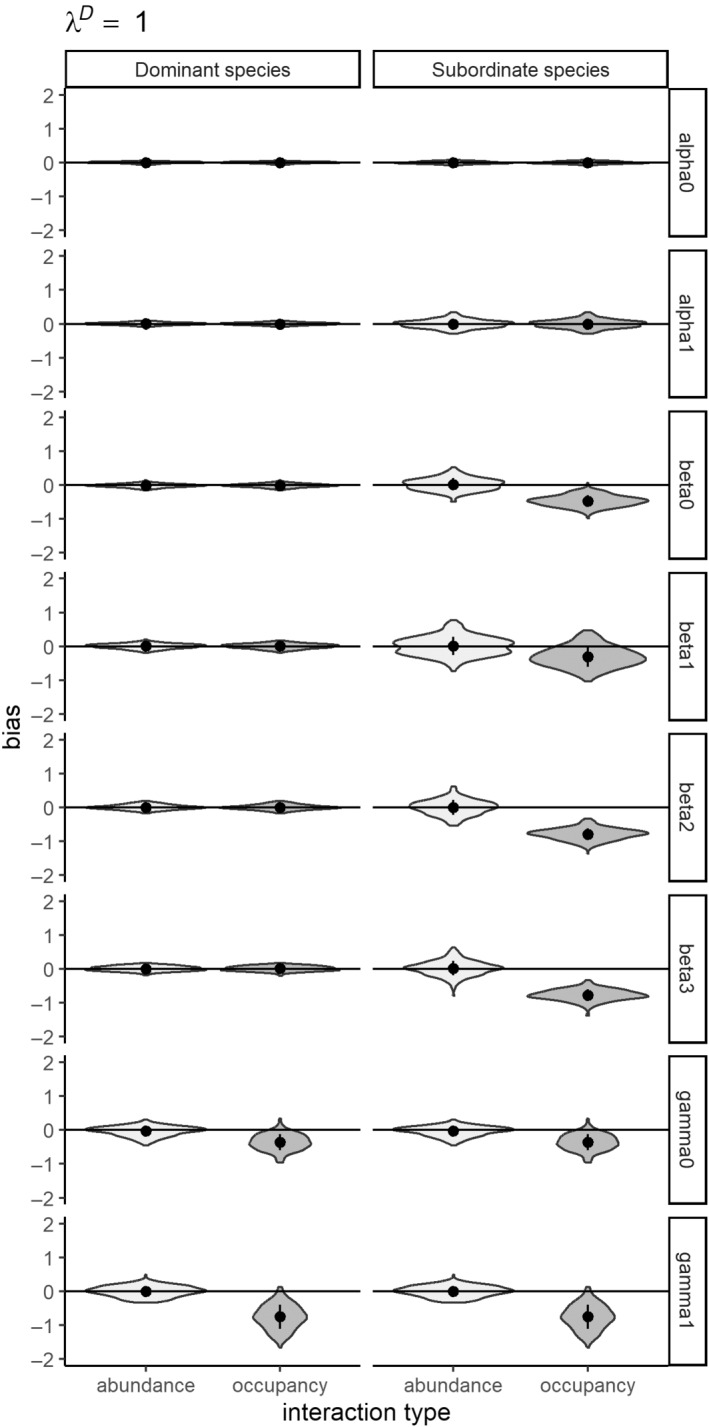
Violin plots showing relative error of parameter estimates from simulation study I (a comparison of modeling species interactions as occupancy‐ vs. abundance‐mediated) which compares modeling interactions as a function of abundance to modeling interactions as function of occupancy states for a moderately abundant dominant species (λ^*D*^ = 1).

**FIGURE 5:**
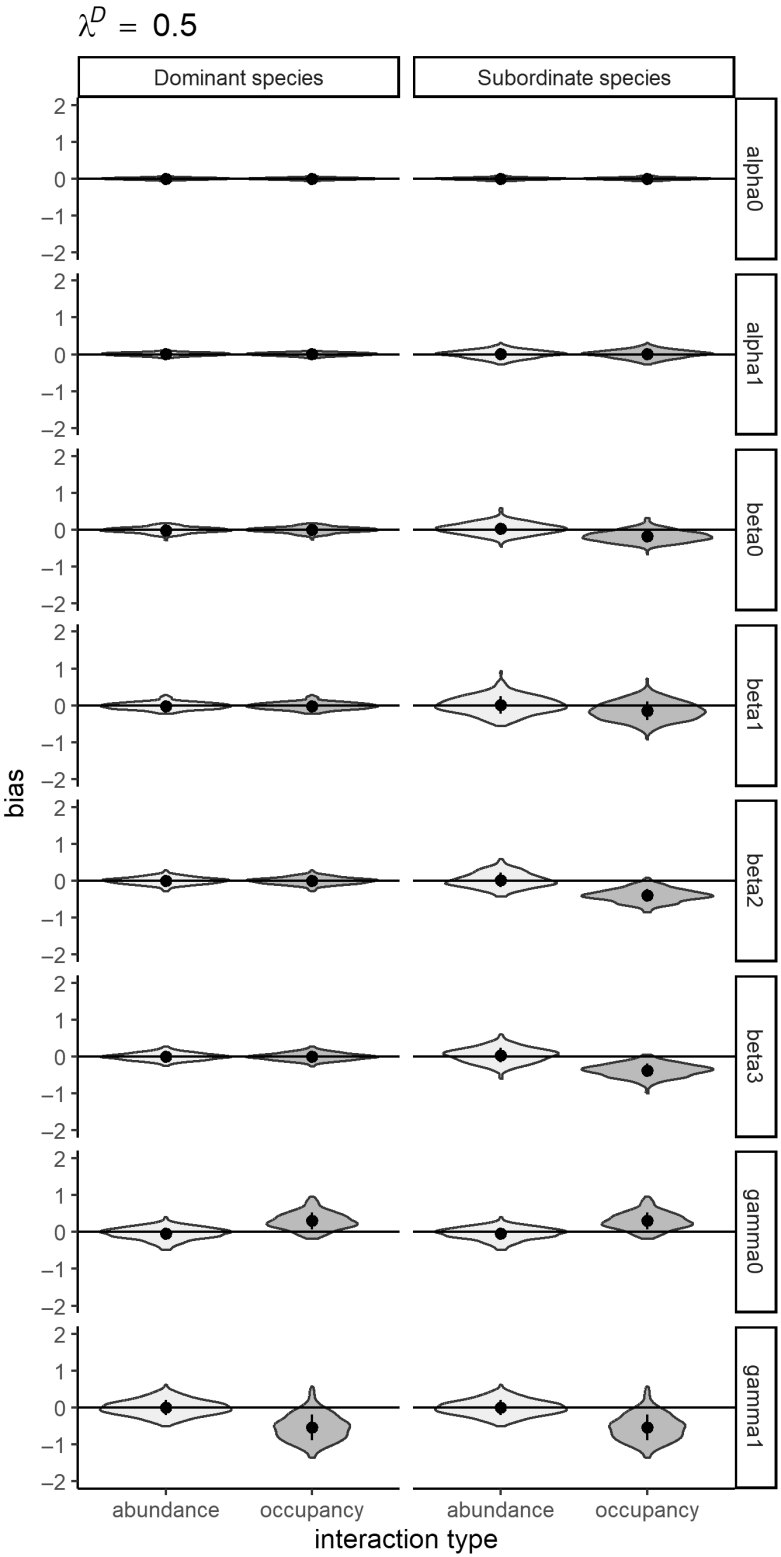
Violin plots showing relative error of parameter estimates from simulation study I (a comparison of modeling species interactions as occupancy‐ vs. abundance‐mediated) which compares modeling interactions as a function of abundance to modeling interactions as function of occupancy states for a low abundance dominant species (λ^*D*^ = 0.5).

The first simulation study demonstrates the importance of modeling species interactions as a function of abundance. All models across all three abundance scenarios converged at sufficiently high rates (see Appendix S1: Table S2). Modeling abundance‐mediated interactions as a function of occupancy results in significant errors in magnitude of the interaction parameter estimates (Type M errors, see Appendix S1: Table S3; Figures 3, 4, 5). While the direction of the interaction term is correctly estimated (no Type S errors), the magnitude of the interaction is overestimated at high abundances (λ^*D*^ = 1–2) and underestimated at lower abundances (λ^*D*^ = 0.5). We observed a reduction in the Type M errors in parameter estimates along with decreasing values of λ^*D*^ (dominant species abundance; Figures 3, 4, 5); nonetheless, error remains high (0.30) even at lower abundances (λ_*D*_ = 0.5). The abundance‐mediated interaction models provided unbiased estimates of all parameters with acceptable coverage in all scenarios (see Appendix S1: Table S3; Figures 3, 4, 5), while the occupancy‐mediated interaction model parameter estimates had Type M errors in all scenarios (see Appendix S1: Table S3).

### Simulation study II: Two‐species simulation with spatially variable complexity and variable sampling effort

**FIGURE 6:**
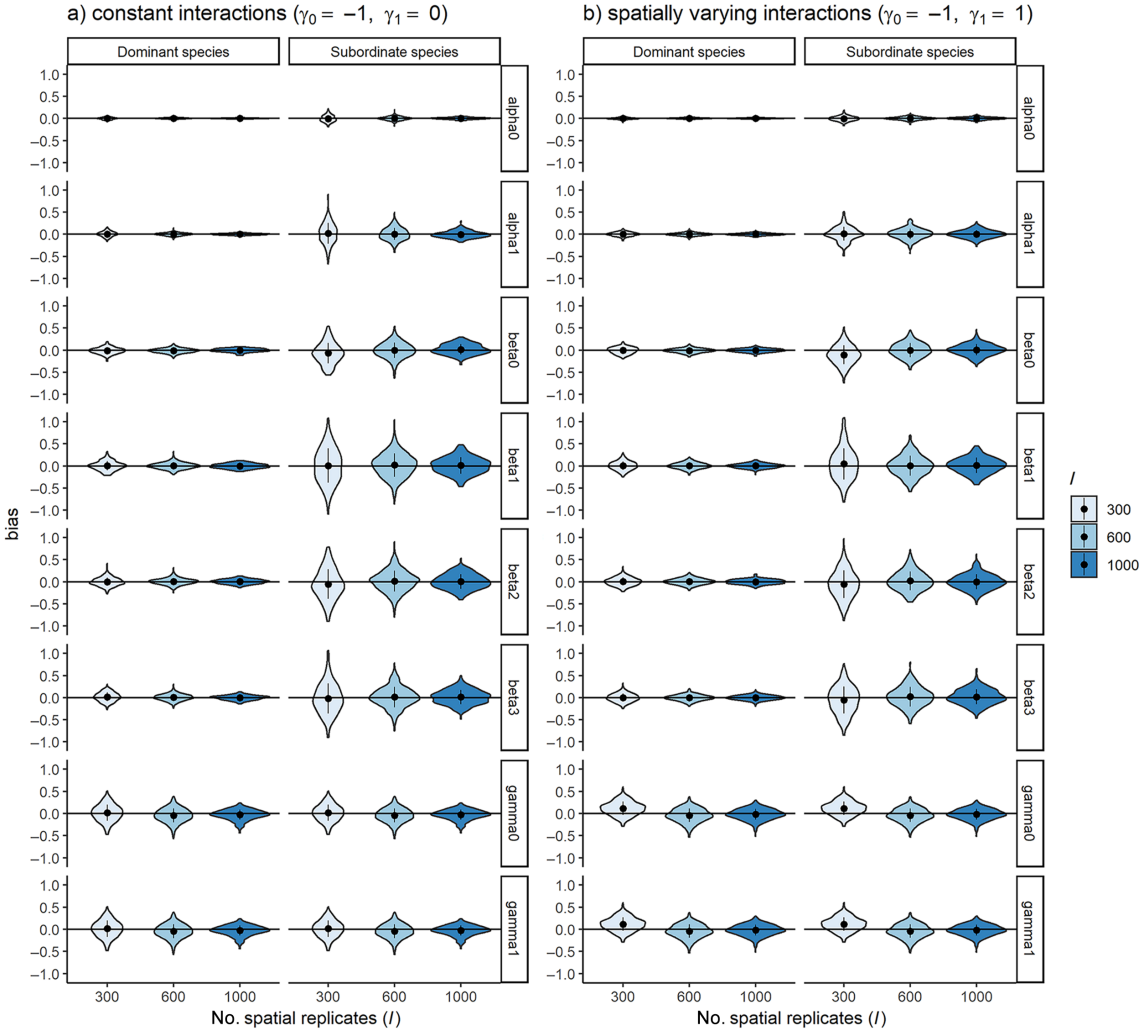
Violin plots showing relative error of parameter estimates from a two species abundance‐mediated interaction model with (a) constant interaction terms (γ_0_ = −1, γ_1_ = 0) and (b) spatially varying interaction terms (γ_0_ = −1, γ_1_ = 1) in simulation scenarios which considered different sampling efforts (*I* = 300, 600, 1000).

For the second simulation study (two species model with variable sampling sites, occasion numbers, and model complexity), unbiased parameter estimates with acceptable coverage were observed for all constant interaction models (i.e., γ_0_ = −1, γ_1_ = 0), even with the lowest number of sites and sampling occasions explored (*I* = 300, *J* = 4; see Figure 6a and Appendix S1: Table S4). However, the sampling requirements were far higher when considering spatially varying interactions (i.e., γ_0_ = −1, γ_1_ = 1; see Figure 6b and Appendix S1: Table S5). For spatially varying interactions, we observed biased parameter estimates in all simulation scenarios with less than 600 sites and 10 occasions. In scenarios with 1000 sites and four or more survey occasions, all parameters were unbiased, with acceptable coverage (see Figure 6 and Appendix S1: Table S5), with 600 sites; bias in the interaction term estimates was variable dependent on the number of sampling occasions (with *J* = 10, estimates were unbiased; see Appendix S1: Table S5).

### Simulation study III: Three‐species simulation with constant interaction terms and variable detection probabilities

**FIGURE 7:**
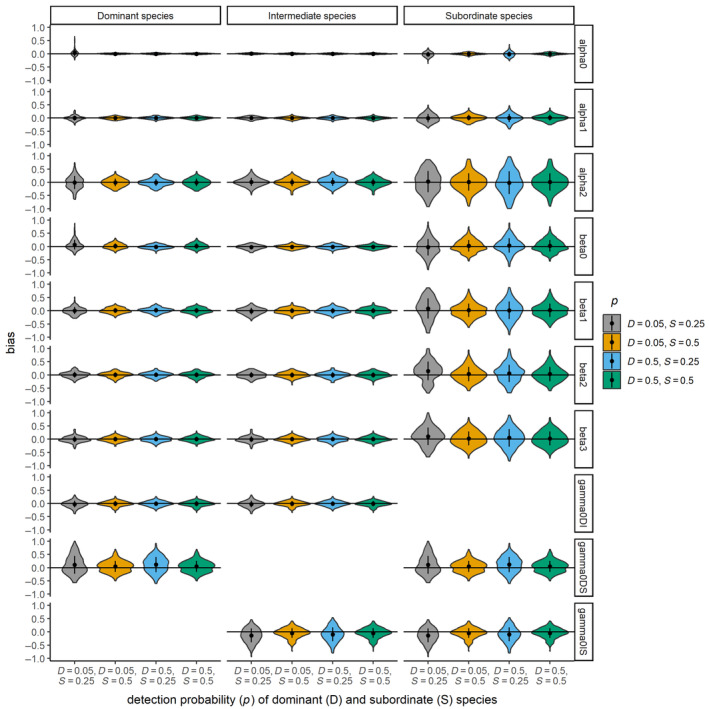
Violin plots showing relative error of parameter estimates from a three‐species abundance‐mediated interaction model with constant interaction terms in simulation study III which considered three species interactions with both high and low detection probabilities for the dominant and subordinate species positions.

In the third simulation study (three‐species model with constant interactions and variable detection probabilities), unbiased estimates of all parameters with acceptable coverage were achieved above a threshold of detection probabilities (*r* ^*D*^ ≥ 0.1, *p* ^*S*^ ≥ 0.5; see Appendix S1: Table S6 and Figure 7). In the cases where a single species had a low mean detection probability, some parameter estimates remain unbiased (see Appendix S1: Table S3); however, interaction terms conditional on the species with low detection probability (i.e., γ_0_^*I‐S*^ and γ_0_^*D‐S*^ when the subordinate species has low detection probability) were biased, but, coverage remained above acceptable levels (see Appendix S1: Table S3). Finally, when multiple species considered have low detection probabilities (i.e., *r* ^*D*^ = 0.05, and *p* ^*S*^ = 0.25), most parameter estimates were biased, and coverage was below acceptable levels (see Appendix S1: Table S7).

### Case study: Intraguild interactions between coyotes, fishers, and American martens

**FIGURE 8:**
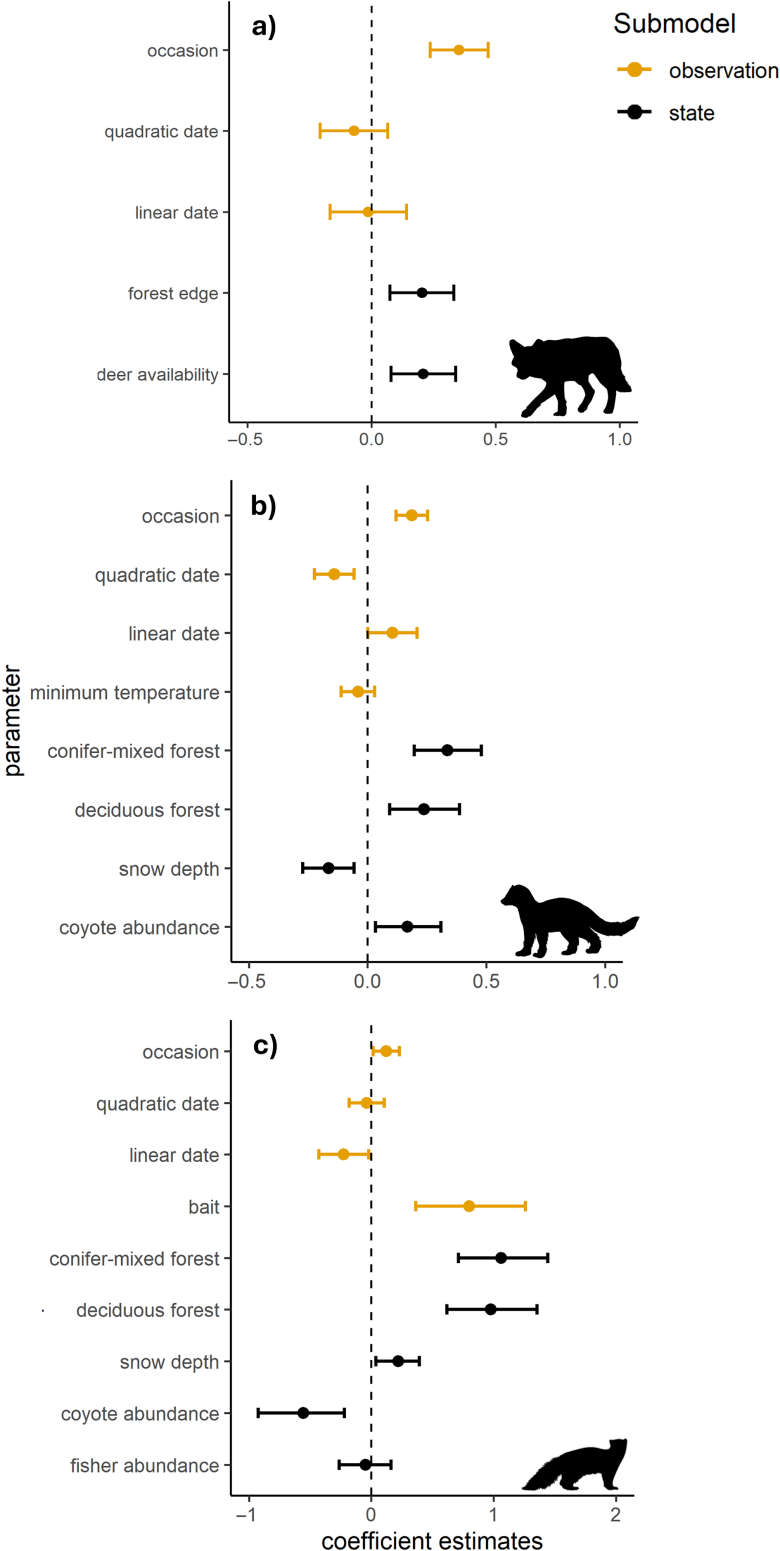
Posterior coefficient estimates with 95% credible intervals for (a) coyote (*Canis latrans*), (b) fisher (*Pekania pennanti*), and (c) marten (*Martes americana*) from three‐species abundance‐mediated interaction model. Illustrations created by Margot Michaud and Gabriela Palomo‐Munoz available under CC0 1.0 Universal Public Domain Dedication licenses and CC BY‐NC 3.0 Attribution‐Non‐Commercial licenses; illustrations were not modified.

All three posterior predictive checks indicated no evidence of lack of fit and all parameters displayed gradual mixing with $\hat{r}$ values <1.1 (see Appendix S1: Table S10 and Figures S4–S7). We found a negative effect of coyote abundance on marten abundance (γ_0_^C‐M^ = −0.55, 95% CI = −0.92 to −0.22; Figure 8c) and a positive effect of coyote abundance on fisher abundance (γ_0_^C‐F^ = 0.17, 95% CI = 0.03–0.31; Figure 6b). There was no effect of fisher abundance on marten abundance (γ_0_^F‐M^ = −0.05, 95% CI = −0.26 to 0.16). Coyote abundance was positively associated with forest edge (β = 0.20, 95% CI = 0.07–0.33) and deer availability (β = 0.21, 95% CI = 0.07–0.34; Figure 8a). In addition to being positively associated with coyote abundance, fisher abundance was positively associated with deciduous forest (β = 0.24, 95% CI = 0.09–0.39), coniferous and mixed forest (β = 0.34, 95% CI = 0.20–0.48), and negatively associated with snow depth (β = −0.16, 95% CI = −0.27 to −0.06; Figure 8c). Finally, marten abundance was positively associated with deciduous forest (β = 0.98, 95% CI = 0.62–1.35), coniferous and mixed forest (β = 1.06, 95% CI = 0.71–1.44), and to a lesser extent, snow depth (β = 0.22, 95% CI = 0.04–0.40).

## DISCUSSION

We demonstrate the critical importance of accounting for abundance‐mediated interactions, a fundamental aspect commonly overlooked within ecology and studies of species interactions. Through multiple simulation studies, we show errors in interaction parameter estimates and potential misinterpretations arising from the conventional approach of modeling interspecific interactions as a function of occupancy states instead of being mediated by species abundance (as ecological theory predicts all interactions to be). To meet this challenge, we develop and demonstrate an abundance‐mediated interaction model for two or more species for modeling species interactions from detection/non‐detection data. This model represents an advancement over prior methodologies which have been limited by a focus largely on occupancy states of species (e.g., Richmond et al., 2010; Rota et al., 2016; Waddle et al., 2010), have relied on N‐mixture modeling of count data (e.g., Brodie et al., 2018; Clare et al., 2016; Dorazio et al., 2015; Roth et al., 2016), or lacked a submodel for detection (e.g., Hui et al., 2015; Johnson & Sinclair, 2017). Results of the simulation studies demonstrate that all parameters in the abundance‐mediated interaction model are identifiable, and unbiased parameter estimates are possible under a variety of ecological and interaction scenarios, including spatial heterogeneity in interaction terms mediated by environmental covariates and three‐species interaction networks. In a practical application focusing on intraguild interactions among coyotes, fishers, and martens, a comparative analysis between modeling interactions based on a function of occupancy states versus abundance highlights a critical revelation: the abundance‐mediated interaction framework unveils otherwise undetected interactions, such as the observed negative relationship between coyotes and martens. These findings underscore the need to update our modeling approaches for a more comprehensive understanding of ecological systems, emphasizing the pivotal role played by abundance‐mediated interspecific interactions in shaping the dynamics of animal populations. Our framework offers a robust pathway for exploring the intricate mechanisms governing ecological communities, guiding more accurate inferences and predictions concerning species dynamics within diverse ecosystems.

We demonstrate via simulation that unbiased parameter estimates of interaction terms are possible, not only for two species models but three species models as well. These simulations demonstrate the potential of this model to reveal and produce important inference into species interactions beyond the scope of pairwise co‐occurrence approaches. We demonstrate the model's potential using a variety of formulations in both two‐ and three‐ species contexts, this multispecies framework provides the possibility for inference into a range of complex interaction networks using a variety of state variables including tritrophic cascades (Terborgh & Estes, 2010), disease‐mediated competition (Slade et al., 2023), and predator‐mediated competition (Twining, Lawton, et al., 2022), while accounting for imperfect detection (see Appendix S1: Applications of abundance‐mediated interaction framework in various ecological contexts with conceptual diagrams and example code).

In our coyote‐fisher‐marten case study, we identify negative abundance‐mediated interactions between coyotes and martens, as hypothesized by Jensen and Humphries (2019). Notably, these interactions were not detected previously when only considering occupancy states of the species within a co‐occurrence framework (Twining, Brazeal, et al., 2024). We see from the first simulation study that the suitability of modeling occupancy‐mediated versus abundance‐mediated interactions depends on system specifics, with key consideration of species density, social organization, and study design. For low density species that establish territories to the exclusion of conspecifics (where site level abundance is likely to vary from 0 to 1), we expect a consensus in inference between modeling interactions as a function of occupancy versus as a function of abundance, as they are *de facto* equivalent. Nonetheless, a situation where species abundance (*N*) only varies from 0 to 1 is improbable to occur in the real world, even for very low‐density species. Thus, for most species, modeling abundance‐mediated interactions as a function of occupancy status will lead to error in parameter estimates. This is demonstrated in our simulation study where extreme Type M errors were still present in our estimates from modeling occupancy states even at our lowest abundances considered (λ = 0.5). This is because in abundance‐mediated interactions, the interaction coefficient estimated is relative to a single unit change in *N*; however, in the binary states of occupancy, variation in *N* is in practice being modeled as a change between 1 and mean *N*. For species where abundance is likely to vary spatially or temporally, occupancy states alone, are insufficient to capture information about interaction frequencies. In such scenarios, models that consider abundance‐mediated interactions provide advantages over co‐occurrence frameworks.

The directionality of interactions has been used in the past to categorize species interactions models (Kéry & Royle, 2021). In the Rota et al. (2016) model, which has the capability to consider more than two species, interactions are symmetrical. While held up as an improvement on previous iterations, this can lead to difficulty in ecological interpretation of interaction parameters when more than two species are being considered (i.e., third order models). In two‐species variations, the only difference between interpretation of symmetrical versus asymmetrical models is when knowledge about the system is applied (*a priori* in the abundance‐mediated interaction models presented here, and post hoc in Rota et al. co‐occurrence models). But, in three‐species systems, the directional approach demanded by abundance‐mediated interaction models, which requires *a priori* hypotheses and explicit specification of processes within the system of interest, provides clarity when interpreting parameter estimates, not present in third order co‐occurrence models.

The abundance‐mediated interaction model presented here is sensitive to detection probabilities of the species considered. This aligns with the first law of capture–recapture that accurate estimation of parameters becomes difficult when *p* is small (Kéry & Royle, 2016; Royle et al., 2014). Previous simulations have shown considerable variation in quality of N‐mixture model estimates, dependent on the combination of sites (*I*), visits (*J*), and detection probability (*p*; Kéry & Royle, 2016). When modeling unmarked individuals using N‐mixture or R–N models, low detection probabilities lead to higher uncertainty in the latent state parameter (*N*_*i*_). In abundance‐mediated interaction models, uncertainty in *N* can result in greater uncertainty in interaction terms and slope parameter estimates for submodels conditional on these estimates (e.g., intermediate and subordinate species). As such, we observe greater uncertainty in the species estimates that have more conditional parameters (e.g., estimates for the subordinate species which are dependent on the state parameter estimates of both other sub models).

We provide evidence to show that this model framework requires relatively large datasets to provide unbiased inference into interactions between species (300 sites [*I*] × four sampling occasions [*J*] for constant interactions). This was anticipated as when using unmarked detection/non‐detection or count data that lacks information required for the identification of individuals, high sample sizes are typically required to achieve the necessary precision in parameter estimates for a variety of population estimation models (Morin et al., 2022; Twining, McFarlene, et al., 2022). When modeling spatially varying interaction terms, unbiased estimates of the interaction parameters (λ_0_ and λ_1_) required a higher sampling intensity (>600 sites [*I*] with 10 survey occasions [*J*]). Acceptable coverage and negligible bias were achieved with 600 sites and four sampling occasions when considering medium–high detection probabilities and constant interactions between two or three species; however, with low detection probabilities or spatially varying interactions, more spatial replicates and/or sampling occasions are required to achieve unbiased parameter estimates (e.g., 600 sites with 10 surveys). Thus, when planning to use this framework to examine interspecific interactions, researchers must consider the importance of accurate point estimates, and devise a sampling design that explicitly considers expected detection probabilities and parameters of interest (e.g., constant vs. spatially varying interactions) and scale the sampling effort (sites [*I*] × visits [*J*]) accordingly. Simulations can be used to devise appropriate sampling designs for different species and ecological contexts of interest, while sampling requirements can be minimized by devising survey methodologies that result in high detection probabilities for the target species (e.g., *r* ≥ 0.1, *p* ≥ 0.5). For example, as seen in the field methods developed for Fuller et al. (2016) using stations baited with beaver for fishers (*P. pennanti*), Evans et al. (2019) using multiple cameras and baited stations for marten (*M. americana*), and Gould et al. (2019) which used cow blood/fish emulsion mixes to achieve high detection probabilities for black bear (*Ursus americanus*). Where additional data sources that are more directly informative about abundance than unmarked detection/non‐detection data are available, for example, distance sampling data, or individual identity data, these data could be integrated into the model. This integration could significantly improve model performance and be achieved simply by using an additional observation model (e.g., that of a standard distance sampling model; Kéry et al., 2024 or a simple SCR model; Royle et al., 2014), with a shared abundance state.

In our pursuit of understanding the dynamics of abundance‐mediated interactions, we have demonstrated the need to update our approaches to modeling species interactions. Specifically, we show that if the objective is to produce robust inference into species interactions, we must consider abundance when we estimate interaction terms between species. To meet this need, we have developed and demonstrated an abundance‐mediated interaction framework for inference into interspecific interactions. Leveraging detection/non‐detection from unmarked populations (with the potential to supplement with more information rich data when available), this framework serves as a flexible tool for deriving insights into ecological communities. We highlight the necessity for consideration of model specifications, with careful consideration of sampling design and detection probabilities required. Nonetheless, the flexible structure of this framework allows the model to be modified to represent a large variety of ecological communities and interaction networks and accommodate a variety of data types. Thus, it emerges as an applicable tool in a wide range of real‐world conservation and management contexts, expanding the scope of species interaction modeling, enabling insights that surpass the limitations of traditional pairwise co‐occurrence approaches. The novel abundance‐mediated interaction framework introduced herein offers a gateway to explore complex interaction networks, encompassing scenarios such as tri‐trophic cascades, mesopredator release, disease‐mediated competition, and predator‐mediated competition.

## CONFLICT OF INTEREST STATEMENT

The authors declare no conflicts of interest.

## Supporting information


Appendix S1.

## Data Availability

Code and data simulators (Twining, Fuller, et al., 2024a) are available in Zenodo at https://doi.org/10.5281/zenodo.10724748. All associated data (Twining, Fuller, et al., 2024b) are available in Dryad at https://doi.org/10.5061/dryad.2bvq83bz2.
