# Supplementary material for: Abundance‐mediated species interactions
Source: Ecology. 2024 Dec 5;106(1):e4468. doi: 10.1002/ecy.4468 (PMC11725697; doi:10.1002/ecy.4468)
Supplement: Supplementary file 1 — Appendix S1. [file ECY-106-e4468-s001.pdf]

Appendix S1

Abundance-mediated species interactions

Joshua P. Twining[1,2]*, Ben C. Augustine[3], J. Andrew Royle[4], Angela K. Fuller[5]

Ecology

Applications of abundance-mediated interaction framework in various ecological contexts with conceptual diagrams and example code

Disease-mediated competition

The model framework could be applied to a wide range of systems and case studies, for example, where there is interest in examining the role of disease-mediated competition in driving system dynamics. Consider a conceptual example where a target population is limited, and there are competing hypotheses around factors driving said population limitation. This could be a case with two competitively linked species, where the dominant competitor is a reservoir host for a range of parasites and pathogens which impact the rarer target species. Understanding the relative impacts of direct vs. disease mediated competition in this system would be critical to managing it to effectively conserve the target species, and the model could be formulated for doing so. In this model iteration we estimate the abundance of the abundant/dominant species using a RN formulation, and the occupancy of the pathogen/parasite and the rarer subordinate species. See Figure S1 for an example using muskoxen (*Ovibos moschatus sspp.)*, nematodes (*Ostertagia gruehneri)*, and caribou (*Rangifer tarandus sspp.)* in the Artic Tundra as a motivating case. Markov Chain Monte Carlo (MCMC) samplers and a data simulator for this model formulation have been developed and are available on Zenodo: https://zenodo.org/records/10724748.

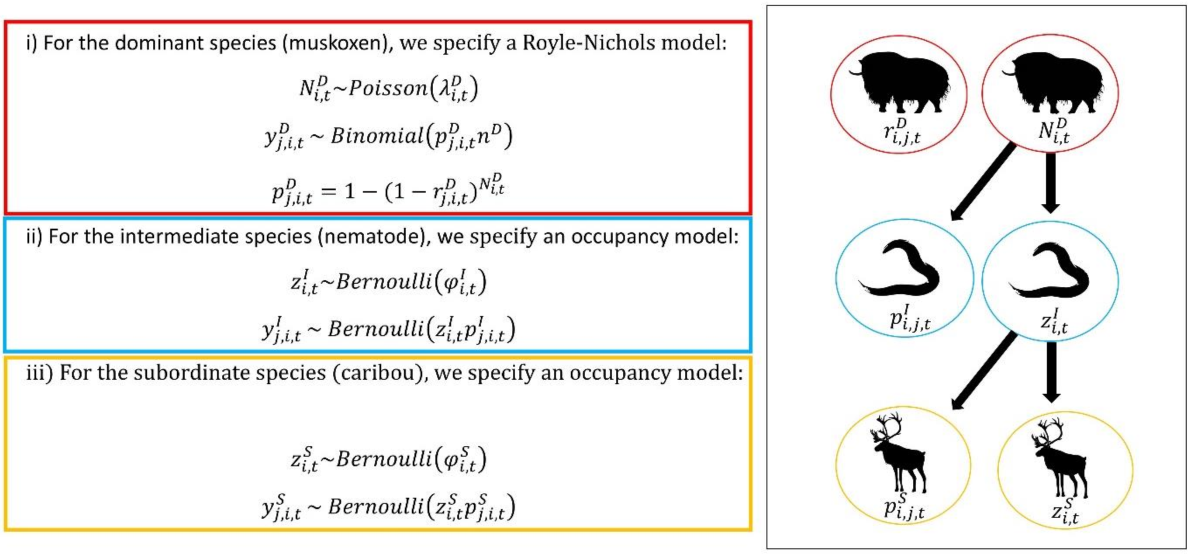

Figure S1. A conceptual diagram of a disease-mediated competition scenario where Muskoxen (*Ovibos moschatus sspp.)* are the dominant species acting as a reservoir host for a pathogenic abomasal nematode (*Ostertagia gruehneri*), which negatively impacts the subordinate competitor, the caribou (*Rangifer tarandus sspp.).* Illustrations by Margot Michaud, Jake Warner and Laura Barbero-Palacios available under CC0 1.0 Universal Public Domain Dedication licenses, illustrations were not modified.

Tri-trophic cascades

Another ecological phenomenon of wide conservation and management interest is that of indirect interactions between trophic levels and species, which can structure ecosystems and drive community outcomes. Tri-trophic cascades specifically involve three species, where the dominant species typically preys upon an intermediate species or consumer, releasing another tertiary prey species or resource from limitation by the consumer/intermediate species. These scenarios are of high applied interest in the context of predator removal, expansion, and reintroductions. For example, the extirpation of large carnivores from the British Isles, the recovery of tigers (*Panthera tigris*) in Nepal, or the reintroduction of wolves (*Canis lupus)* into various locations across the continental United States. This model can be adapted to formally examine these types of cascading interactions statistically. In this model, we estimate the abundance of all three species using an RN formulation in each submodel. See Figure S2 for an example using the exemplar case of trophic

cascades involving otters (*Enhydra lutris)*, sea urchins (*Strongylocentrotus sp*.), and kelp (*Phaeophyta sp.)*. MCMC samplers and a data simulator for this model formulation have been developed and are available on Zenodo: https://zenodo.org/records/10724748.

It is worth noting here, that indirect interactions will have to be interpreted from the direct parameter estimates. For example, in our otter-urchin-kelp case study (see Figure S2), if an interaction was specified between otters and kelp ($\gamma_{D\text{-}S}$), in addition to between otters and urchins ($\gamma_{D\text{-}I}$), and urchins and kelp ($\gamma_{I\text{-}S}$), the former interaction term estimated between otters and kelp would be 0. This is because the indirect impacts of otters on kelp through limitation of urchins are already accounted for in the direct interactions between the dominant and intermediate ($\gamma_{D\text{-}I}$), and the intermediate and subordinate species ($\gamma_{I\text{-}S}$). Nonetheless, the estimation of these direct interactions in systems will enable users to infer the indirect interactions in the system with precision.

i) For the dominant species (otter), we specify a Royle-Nichols model:

$$N_{i,t}^{D} \sim Poisson(\lambda_{i,t}^{D})$$

$$y_{i,j,t}^{D} \sim Binomial(p_{i,j,t}^{D} K^{D})$$

$$p_{i,j,t}^{D} = 1 - (1 - r_{i,j,t}^{D})^{N_{i,t}^{D}}$$

ii) For the intermediate species (urchin), we specify a Royle-Nichols model:

$$N_{i,t}^{I} \sim Poisson(\lambda_{i,t}^{I})$$

$$y_{i,j,t}^{I} \sim Binomial\ (p_{i,j,t}^{I} K^{I})$$

$$p_{i,j,t}^{I} = 1 - (1 - r_{i,j,t}^{I})^{N_{i,t}^{I}}$$

iii) For the subordinate species (producer), we specify a Royle-Nichols model:

$$N_{i,t}^{S} \sim Poisson(\lambda_{i,t}^{S})$$

$$y_{i,j,t}^{S} \sim Binomial(p_{i,j,t}^{S} K^{S})$$

$$p_{i,j,t}^{S} = 1 - (1 - r_{i,j,t}^{S})^{N_{i,t}^{S}}$$

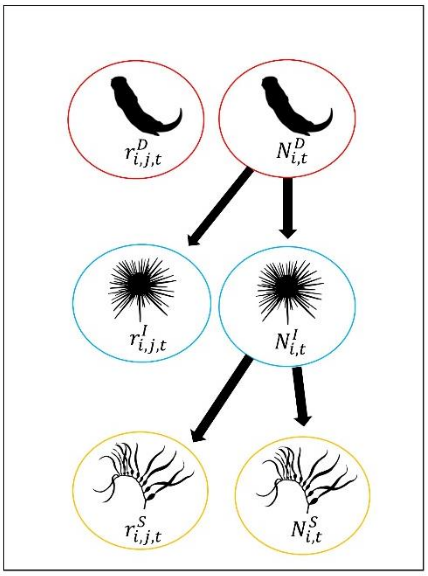

Figure S2. A conceptual diagram of a trophic cascade scenario where sea otters (*Enhydra lutris*) are suppressing sea urchins (*Strongylocentrotus sp*.), which in turn are suppressing kelp (*Phaeophyta sp.).* Illustrations by Margot Michaud, Guillaume Dera, and Harold N Eyster, available under CC0 1.0 Universal Public Domain Dedication licenses, illustrations were not modified.

Predator mediated competition.

A third frequently encountered and ecologically critical interaction scenario are cases whereby a shared predator structures and impacts outcomes in a system between two competitively linked prey species. Such scenarios are ubiquitous in ecological systems, but those involving invasive species of are particularly high management and conservation importance. For example, the interactions between the recovering native pine marten (*Martes martes*), and its competitively linked prey, the invasive gray squirrel (*Sciurus carolinensis)*, and the native red squirrel (*Sciurus vulgaris*) in Europe, or the interactions between the planktivorous capelin (*Mallotus villosus)* and two zooplankton groups (krill and copepods) in the Barents Sea. This model would be highly suitable to such scenarios, in our example model, we develop a model whereby we estimate the occupancy of the dominant species and abundance of the intermediate and subordinate species using Royle-Nichols models. See Figure S3 and associated code for an example using the interactions between the recovering native pine marten (*Martes martes*), and its competitively linked prey, the invasive gray squirrel (*Sciurus carolinensis)*, and the native red squirrel (*Sciurus vulgaris*) in Europe as a motivating case study. Markov Chain Monte Carlo (MCMC) samplers and a data simulator for this model formulation have been developed and are available on Zenodo: https://zenodo.org/records/10724748.

i) For the dominant species (pine marten), we specify an occupancy model:

$$z^D_{i,t} \sim Bernoulli(\varphi^D_{i,t})$$

$$y^D_{i,j,t} \sim Bernoulli(z^D_{i,t} p^D_{i,j,t})$$

ii) For the intermediate species (grey squirrel), we specify a Royle-Nichols model:

$$N^I_{i,t} \sim Poisson(\lambda^I_{i,t})$$

$$y^I_{i,j,t} \sim Binomial\ (p^I_{i,j,t} K^I)$$

$$p^I_{i,j,t} = 1 - (1 - r^I_{i,j,t})^{N^I_{i,t}}$$

iii) For the subordinate species (red squirrel), we specify a Royle-Nichols model:

$$N^S_{i,t} \sim Poisson(\lambda^S_{i,t})$$

$$y^S_{i,j,t} \sim Binomial(p^S_{i,j,t} K^S)$$

$$p^S_{i,j,t} = 1 - (1 - r^S_{i,j,t})^{N^S_{i,t}}$$

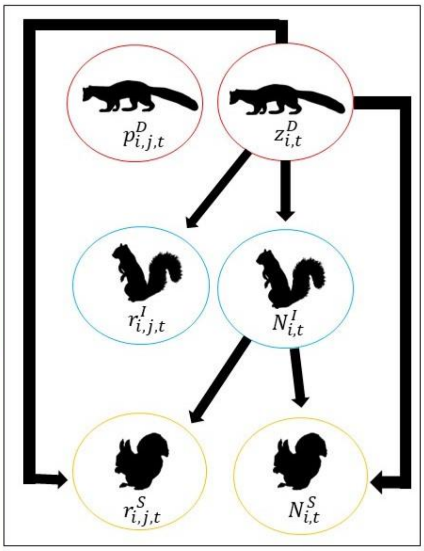

Figure S3. A conceptual diagram of a predator-mediated competition scenario where pine martens *(Martes martes)* are predating both grey (*Sciurus carolinensis)* and red squirrels (*Sciurus vulgaris)*,

two competitively linked prey species, in which the invasive grey squirrel is the dominant competitor. Illustrations by Ferran Sayol and Andy Wilson available under CC0 1.0 Universal Public Domain Dedication licenses, illustrations were not modified.

Uninformative priors for simulation studies

Table S1. Uninformative tau priors used in simulation studies I, II and III.

Parameter	Prior
α_{0D}	Logistic(0, 1)
α_{1D}	Normal(0, 0.1)
α_{2D}	Normal(0, 0.1)
β_{0D}	Normal(0, 0.1)
β_{1D}	Normal(0, 0.1)
β_{2D}	Normal(0, 0.1)
β_{3D}	Normal(0, 0.1)
β_{0S}	Logistic(0, 1)
α_{0S}	Normal(0, 0.1)
α_{1S}	Normal(0, 0.1)
α_{2S}	Normal(0, 0.1)
β_{0S}	Logistic(0, 0.1)
β_{1S}	Normal(0, 0.1)
β_{2S}	Normal(0, 0.1)
β_{3S}	Normal(0, 0.1)
β_{0S}	Normal(0, 0.1)
λ_0	Normal(0, 0.2)
λ_1	Normal(0, 0.2)

Simulation study I – a comparison of modelling species interactions as occupancy- vs. abundance-mediated

Simulation code can be found here: https://zenodo.org/records/10724748

Table S2. Convergence rates for each simulation scenario from a total of 250 datasets simulated for each of the six different parameter combinations.

Parameter combination	Converged (%)
$\gamma_0 * z^D$, $\lambda^D = 2$ (occupancy mediated, high mean abundance)	91.6
$\gamma_0 * N^D$, $\lambda^D = 2$ (abundance mediated, high mean abundance)	93.2
$\gamma_0 * z^D$, $\lambda^D = 1$ (occupancy mediated, medium mean abundance)	97.6
$\gamma_0 * N^D$, $\lambda^D = 1$ (abundance mediated, medium mean abundance)	97.2
$\gamma_0 * z^D$, $\lambda^D = 0.5$ (occupancy mediated, low mean abundance)	99.2
$\gamma_0 * N^D$, $\lambda^D = 0.5$ (abundance mediated, low mean abundance)	99.2

Table S3. Relative bias and coverage of each parameter from 250 datasets simulated from each of the 27 different parameter combinations.

Parameter combination	Parameter	Relative Bias	Coverage (%)
$\gamma_0 * z^D$, $\lambda^D = 2$ (occupancy mediated, high mean abundance	γ_0	-0.52	54.59
	γ_1	-0.92	43.67
	β_0^D	-0.02	93.01
	β_0^S	-0.94	63.11
	β_1^D	0.00	97.38
	β_1^S	-0.49	97.38
	β_2^D	0.00	95.20
	β_2^S	-1.18	95.20
	β_3^D	0.00	92.58
	β_3^S	-1.20	92.58
	α_0^D	0.01	94.76
	α_0^S	0.00	94.32
	α_1^D	0.00	94.76
	α_1^S	0.01	94.76
	α_2^D	0.00	93.45
	α_2^S	-0.03	93.45
	γ_0	-0.05	92.27
	γ_1	-0.02	95.71

$\gamma_0 * N^D$, $\lambda^D = 2$ (abundance mediated, high mean abundance)	β_0^D	-0.02	93.56
	β_0^S	0.04	93.99
	β_1^D	0.00	97.42
	β_1^S	0.05	97.42
	β_2^D	0.00	95.71
	β_2^S	0.03	95.71
	β_3^D	0.00	91.42
	β_3^S	-0.01	91.42
	α_0^D	0.01	93.56
	α_0^S	0.00	94.42
	α_1^D	0.01	94.85
	α_1^S	0.01	94.85
	α_2^D	0.00	93.56
	α_2^S	-0.01	93.56
$\gamma_0 * z^D$, $\lambda^D = 1$ (occupancy mediated, medium mean abundance)	γ_0	-0.36	65.16
	γ_1	-0.75	42.21
	β_0^D	-0.01	95.49
	β_0^S	-0.47	27.46
	β_1^D	0.00	96.31
	β_1^S	-0.31	96.31
	β_2^D	0.01	93.03
	β_2^S	-0.78	93.03
	β_3^D	0.01	96.72
	β_3^S	-0.77	96.72
	α_0^D	0.00	95.49
	α_0^S	0.00	94.67
	α_1^D	0.00	96.31
	α_1^S	0.00	96.31
	α_2^D	0.00	96.72
	α_2^S	0.03	96.72
$\gamma_0 * N^D$, $\lambda^D = 1$ (abundance mediated, medium mean abundance)	γ_0	-0.03	93.42
	γ_1	0.00	97.94
	β_0^D	-0.01	94.65
	β_0^S	0.02	95.88
	β_1^D	0.00	95.88
	β_1^S	0.01	95.88
	β_2^D	0.01	93.42
	β_2^S	0.01	93.42
	β_3^D	0.00	96.71
	β_3^S	0.02	96.71
	α_0^D	0.00	95.06
	α_0^S	0.00	95.06
	α_1^D	0.00	95.47
	α_1^S	0.00	95.47
	α_2^D	0.00	96.71

	α_2^{S}	0.02	96.71
$\gamma_0 * z^{D}$, $\lambda^{D} = 0.5$ (occupancy mediated, low mean abundance)	γ_0	0.30	76.61
	γ_1	-0.54	62.10
	β_0^{D}	0.00	91.13
	β_0^{S}	-0.17	79.03
	β_1^{D}	-0.02	94.35
	β_1^{S}	-0.14	94.35
	β_2^{D}	0.00	95.56
	β_2^{S}	-0.39	95.56
	β_3^{D}	-0.01	94.35
	β_3^{S}	-0.38	94.35
	α_0^{D}	0.00	98.79
	α_0^{S}	0.00	96.37
	α_1^{D}	0.00	96.37
	α_1^{S}	0.00	96.37
	α_2^{D}	0.00	95.97
	α_2^{S}	-0.04	95.97
$\gamma_0 * N^{D}$, $\lambda^{D} = 0.5$ (abundance mediated, low mean abundance)	γ_0	-0.04	93.95
	γ_1	-0.01	94.76
	β_0^{D}	-0.01	91.13
	β_0^{S}	0.03	96.37
	β_1^{D}	-0.02	93.95
	β_1^{S}	0.01	93.95
	β_2^{D}	0.00	95.16
	β_2^{S}	0.02	95.16
	β_3^{D}	0.00	94.35
	β_3^{S}	0.03	94.35
	α_0^{D}	0.00	97.98
	α_0^{S}	0.00	95.56
	α_1^{D}	0.00	95.97
	α_1^{S}	0.00	95.97
	α_2^{D}	0.00	95.97
	α_2^{S}	-0.04	95.97

Simulation study II – two-species simulation with spatially varying interaction terms and variable sampling effort

Simulation code can be found here: : https://zenodo.org/records/10724748

Table S4. Convergence rates for each simulation scenario from a total of 250 datasets simulated for each of the twelve different parameter combinations.

Parameter combination	Converged (%)
I=300, J = 4, γ_0 = -1, γ_1 = 0	94.4
I=300, J = 10, γ_0 = -1, γ_1 = 0	96.4
I=300, J = 4, γ_0 = -1, γ_1 = 1	67.2
I=300, J = 10, γ_0 = -1, γ_1 = 1	76.4
I=600, J = 4, γ_0 = -1, γ_1 = 0	96.4
I=600, J = 10, γ_0 = -1, γ_1 = 0	100
I=600, J = 4, γ_0 = -1, γ_1 = 1	99.6
I=600, J = 10, γ_0 = -1, γ_1 = 1	100
I=1000, J = 4, γ_0 = -1, γ_1 = 0	99.6
I=1000, J = 10, γ_0 = -1, γ_1 = 0	98
I=1000, J = 4, γ_0 = -1, γ_1 = 1	100
I=1000, J = 10, γ_0 = -1, γ_1 = 1	100

Table S5. Relative bias and coverage of each parameter from 250 datasets simulated from each of the 27 different parameter combinations.

Parameter combination	Parameter	Relative Bias	Coverage (%)
I=300, J=4, γ_0 = -1, γ_1 = 0	γ_0	-0.01	94.07
	γ_1	-0.04	98.73
	β_0^D	-0.01	96.61
	β_0^S	-0.04	93.64
	β_1^D	0.01	97.46
	β_1^S	0.01	97.46
	β_2^D	0.02	95.34
	β_2^S	0.00	95.34
	β_3^D	0.01	94.49
	β_3^S	0.00	94.49
	α_0^D	0.00	97.03
	α_0^S	0.00	95.76
	α_1^D	0.00	94.92
	α_1^S	0.01	94.92
	α_2^D	0.00	95.34
	α_2^S	0.01	95.34
I=300, J=4, γ_0 = -1, γ_1 = 1	γ_0	0.12	90.48
	γ_1	-0.14	91.67
	β_0^D	-0.01	94.64

	β_0^S	-0.11	90.48
	β_1^D	0.01	94.05
	β_1^S	0.06	94.05
	β_2^D	0.01	96.43
	β_2^S	-0.07	96.43
	β_3^D	0.02	95.83
	β_3^S	-0.04	95.83
	α_0^D	0.00	95.24
	α_0^S	0.00	93.45
	α_1^D	0.00	96.43
	α_1^S	0.01	96.43
	α_2^D	0.01	96.43
	α_2^S	-0.01	96.43
I=300, J=10, γ_0 = -1, γ_1 = 0	γ_0	-0.01	94.07
	γ_1	-0.04	98.73
	β_0^D	-0.01	96.61
	β_0^S	-0.04	93.64
	β_1^D	0.01	97.46
	β_1^S	0.01	97.46
	β_2^D	0.02	95.34
	β_2^S	0.00	95.34
	β_3^D	0.01	94.49
	β_3^S	0.00	94.49
	α_0^D	0.00	97.03
	α_0^S	0.00	95.76
	α_1^D	0.00	94.92
	α_1^S	0.01	94.92
	α_2^D	0.00	95.34
	α_2^S	0.01	95.34
I=300, J=10, γ_0 = -1, γ_1 = 1	γ_0	0.11	90.58
	γ_1	-0.13	91.62
	β_0^D	0.00	96.34
	β_0^S	-0.10	91.10
	β_1^D	0.01	94.24
	β_1^S	0.04	94.24
	β_2^D	0.01	97.91
	β_2^S	-0.04	97.91
	β_3^D	-0.01	96.86
	β_3^S	-0.06	96.86
	α_0^D	0.00	93.19
	α_0^S	0.00	93.19
	α_1^D	0.00	95.81
	α_1^S	0.01	95.81
	α_2^D	0.00	94.76
	α_2^S	0.01	94.76

I=600, J=4, γ_0 = -1, γ_1 = 0	γ_0	-0.07	93.78
	γ_1	0.01	95.85
	β_0^D	-0.02	94.19
	β_0^S	0.02	95.85
	β_1^D	0.00	93.78
	β_1^S	0.02	93.78
	β_2^D	0.00	94.61
	β_2^S	0.03	94.61
	β_3^D	0.01	94.19
	β_3^S	0.03	94.19
	α_0^D	0.00	94.19
	α_0^S	-0.01	95.85
	α_1^D	0.00	94.19
	α_1^S	0.02	94.19
	α_2^D	0.00	95.44
	α_2^S	0.02	95.44
I=600, J=4, γ_0 = -1, γ_1 = 1	γ_0	-0.05	94.80
	γ_1	0.07	93.60
	β_0^D	-0.01	95.60
	β_0^S	0.00	95.60
	β_1^D	0.01	94.40
	β_1^S	0.01	94.40
	β_2^D	0.00	93.60
	β_2^S	0.04	93.60
	β_3^D	0.00	93.20
	β_3^S	0.02	93.20
	α_0^D	0.00	97.20
	α_0^S	0.00	96.80
	α_1^D	0.00	94.80
	α_1^S	0.01	94.80
	α_2^D	0.01	96.80
	α_2^S	-0.03	96.80
I=600, J=10, γ_0 = -1, γ_1 = 0	γ_0	-0.05	94.69
	γ_1	0.00	95.51
	β_0^D	-0.01	92.65
	β_0^S	0.01	94.29
	β_1^D	0.01	94.29
	β_1^S	0.04	94.29
	β_2^D	0.00	93.88
	β_2^S	0.02	93.88
	β_3^D	0.00	96.33
	β_3^S	0.02	96.33
	α_0^D	0.00	95.51
	α_0^S	0.00	94.29
	α_1^D	0.00	94.69

	$\alpha_1{}^S$	0.00	94.69
	$\alpha_2{}^D$	0.00	94.29
	$\alpha_2{}^S$	0.01	94.29
I=600, J=10, γ_0 = -1, γ_1 = 1	γ_0	-0.03	93.60
	γ_1	0.04	92.00
	$\beta_0{}^D$	-0.01	96.00
	$\beta_0{}^S$	0.00	94.00
	$\beta_1{}^D$	0.00	93.60
	$\beta_1{}^S$	0.00	93.60
	$\beta_2{}^D$	0.01	95.60
	$\beta_2{}^S$	0.01	95.60
	$\beta_3{}^D$	-0.01	98.00
	$\beta_3{}^S$	0.02	98.00
	$\alpha_0{}^D$	0.00	93.20
	$\alpha_0{}^S$	0.00	94.40
	$\alpha_1{}^D$	0.00	96.40
	$\alpha_1{}^S$	0.00	96.40
	$\alpha_2{}^D$	0.00	92.40
	$\alpha_2{}^S$	0.00	92.40
I=1000, J=4, γ_0 = -1, γ_1 = 0	γ_0	-0.03	95.63
	γ_1	-0.02	96.07
	$\beta_0{}^D$	-0.01	96.94
	$\beta_0{}^S$	0.01	93.89
	$\beta_1{}^D$	0.00	95.20
	$\beta_1{}^S$	0.01	95.20
	$\beta_2{}^D$	0.00	94.32
	$\beta_2{}^S$	0.00	94.32
	$\beta_3{}^D$	0.01	93.89
	$\beta_3{}^S$	-0.01	93.89
	$\alpha_0{}^D$	0.00	96.51
	$\alpha_0{}^S$	0.00	93.45
	$\alpha_1{}^D$	0.00	97.38
	$\alpha_1{}^S$	-0.01	97.38
	$\alpha_2{}^D$	0.00	93.89
	$\alpha_2{}^S$	0.03	93.89
I=1000, J=4, γ_0 = -1, γ_1 = 1	γ_0	-0.02	93.60
	γ_1	0.03	94.80
	$\beta_0{}^D$	-0.01	94.00
	$\beta_0{}^S$	0.00	92.80
	$\beta_1{}^D$	0.00	95.20
	$\beta_1{}^S$	0.01	95.20
	$\beta_2{}^D$	0.00	96.00
	$\beta_2{}^S$	0.01	96.00
	$\beta_3{}^D$	0.00	95.20
	$\beta_3{}^S$	0.02	95.20

	$\alpha_0{}^D$	0.00	92.40
	$\alpha_0{}^S$	0.00	94.80
	$\alpha_1{}^D$	0.00	96.00
	$\alpha_1{}^S$	0.01	96.00
	$\alpha_2{}^D$	0.00	94.80
	$\alpha_2{}^S$	0.03	94.80
I=1000, J=10, $\gamma_0 = -1$, $\gamma_1 = 0$	γ_0	-0.03	92.65
	γ_1	-0.01	95.92
	$\beta_0{}^D$	0.00	92.65
	$\beta_0{}^S$	0.02	96.73
	$\beta_1{}^D$	0.00	95.51
	$\beta_1{}^S$	0.01	95.51
	$\beta_2{}^D$	0.01	95.51
	$\beta_2{}^S$	0.01	95.51
	$\beta_3{}^D$	0.00	95.10
	$\beta_3{}^S$	0.02	95.10
	$\alpha_0{}^D$	0.00	93.47
	$\alpha_0{}^S$	0.00	94.29
	$\alpha_1{}^D$	0.00	95.92
	$\alpha_1{}^S$	0.00	95.92
	$\alpha_2{}^D$	0.00	94.69
	$\alpha_2{}^S$	0.00	94.69
I=1000, J=10, $\gamma_0 = -1$, $\gamma_1 = 1$	γ_0	-0.02	94.40
	γ_1	0.02	94.00
	$\beta_0{}^D$	0.00	95.60
	$\beta_0{}^S$	0.01	93.20
	$\beta_1{}^D$	0.01	97.60
	$\beta_1{}^S$	0.01	97.60
	$\beta_2{}^D$	0.00	95.20
	$\beta_2{}^S$	0.00	95.20
	$\beta_3{}^D$	0.00	94.40
	$\beta_3{}^S$	0.02	94.40
	$\alpha_0{}^D$	0.00	95.60
	$\alpha_0{}^S$	0.00	92.80
	$\alpha_1{}^D$	0.00	97.20
	$\alpha_1{}^S$	0.01	97.20
	$\alpha_2{}^D$	0.00	94.40
	$\alpha_2{}^S$	0.00	94.40

Simulation study III – three-species simulation with constant interactions and variable detection probabilities

Simulation code can be found here: https://zenodo.org/records/10724748

Table S6. Convergence rates for each simulation scenario from a total of 250 datasets simulated for each of the four different parameter combinations.

Parameter combination	Converged (%)
r^D=0.5, p^S = 0.5	97.6
r^D=0.05, p^S = 0.5	95.2
r^D=0.5, p^S = 0.25	95.2
r^D=0.05, p^S = 0.25	73.2

Table S7. Relative bias and coverage of each parameter from 250 datasets simulated from each of the four different parameter combinations.

Parameter combination	Parameter	Relative Bias	Coverage (%)
r^D=0.5, p^S = 0.5	$\gamma_0^{D\text{-}S}$	0.04	95.90
	$\gamma_0^{I\text{-}S}$	-0.04	91.80
	$\gamma_0^{D\text{-}I}$	-0.01	92.62
	β_0^D	0.02	94.67
	β_0^I	-0.01	93.85
	β_0^S	0.02	94.67
	β_1^D	0.01	95.90
	β_1^I	0.00	95.90
	β_1^S	0.02	93.03
	β_2^D	0.01	94.26
	β_2^I	0.00	94.26
	β_2^S	0.04	93.03
	β_3^D	0.00	96.31
	β_3^I	0.00	94.96
	β_3^S	0.03	93.44
	α_0^D	0.00	96.72
	α_0^I	0.00	94.67
	α_0^S	0.00	93.85
	α_1^D	0.00	95.90
	α_1^I	0.00	94.26
	α_1^S	0.01	95.90
	α_2^D	0.00	95.90
	α_2^I	0.00	96.64
	α_2^S	0.02	96.26
r^D=0.05, p^S = 0.5	$\gamma_0^{D\text{-}S}$	0.09	94.12
	$\gamma_0^{I\text{-}S}$	-0.08	94.12
	$\gamma_0^{D\text{-}I}$	-0.02	94.96

	β_0^D	0.05	93.28
	β_0^I	-0.01	94.54
	β_0^S	-0.06	93.70
	β_1^D	0.01	95.80
	β_1^I	0.00	94.96
	β_1^S	0.02	96.22
	β_2^D	0.01	94.96
	β_2^I	-0.01	94.96
	β_2^S	0.02	93.28
	β_3^D	0.00	94.96
	β_3^I	0.00	94.96
	β_3^S	0.03	96.22
	α_0^D	0.02	92.86
	α_0^I	0.00	97.06
	α_0^S	0.00	93.70
	α_1^D	0.00	96.22
	α_1^I	0.00	93.70
	α_1^S	0.00	94.12
	α_2^D	-0.01	94.96
	α_2^I	-0.01	92.86
	α_2^S	0.03	96.64
r^D=0.5, p^S = 0.25	γ_0^{D-S}	0.12	94.54
	γ_0^{I-S}	-0.10	94.96
	γ_0^{D-I}	-0.01	95.38
	β_0^D	0.03	96.22
	β_0^I	-0.01	93.70
	β_0^S	0.04	94.12
	β_1^D	0.02	94.54
	β_1^I	0.00	94.96
	β_1^S	0.03	95.38
	β_2^D	0.01	94.96
	β_2^I	0.00	94.96
	β_2^S	0.07	94.96
	β_3^D	0.00	94.54
	β_3^I	0.00	94.96
	β_3^S	0.06	94.96
	α_0^D	0.00	91.18
	α_0^I	0.00	94.54
	α_0^S	-0.01	93.28
	α_1^D	0.00	94.54
	α_1^I	0.00	95.38
	α_1^S	0.00	94.96
	α_2^D	-0.01	96.22
	α_2^I	0.01	93.28
	α_2^S	0.00	94.54

r^D=0.05, p^S = 0.25	γ_0^{D-S}	0.12	95.63
	γ_0^{I-S}	-0.14	95.63
	γ_0^{D-I}	-0.03	93.44
	β_0^D	0.07	90.16
	β_0^I	-0.02	92.35
	β_0^S	-0.01	93.44
	β_1^D	0.00	89.07
	β_1^I	-0.02	92.90
	β_1^S	0.10	96.72
	β_2^D	0.01	93.99
	β_2^I	0.00	93.99
	β_2^S	0.16	93.99
	β_3^D	0.00	93.99
	β_3^I	0.00	94.96
	β_3^S	0.12	93.44
	α_0^D	0.04	91.80
	α_0^I	0.00	95.63
	α_0^S	-0.02	93.44
	α_1^D	0.00	95.63
	α_1^I	0.00	95.08
	α_1^S	0.00	97.81
	α_2^D	0.00	96.17
	α_2^I	0.01	92.35
	α_2^S	0.02	96.17

Case study – three species abundance-mediated interactions between coyotes-fishers-marten in northeastern North America

Coyote-fisher-marten abundance mediated interaction model with binomial observation model code can be found here: https://zenodo.org/records/10724748.

To determine drivers of species abundance we considered six landscape covariates that were considered in Twining et al. (2024a), that have been observed to impact occurrence and abundance of the three species (Fuller *et al.* 2016; Jensen & Humphries, 2019; Siren *et al.* 2021). These covariates were related to forest composition (% deciduous forest, % coniferous and mixed forest, and forest edge density (km/km^2)), deer availability (white-tailed deer [*Odocoileus virginianus*] probability of occupancy) and abiotic conditions (average minimum temperature (°C) and average snow depth (mm)). For details on units, description, and source of landscape covariates see Table S8. Deer availability was calculated at a scale relevant to white-tailed deer movement around sampling sites (2km^2, Quinn *et al.* 2013; see below Table S9 and S10 for full details). To explain variation in detection

probability we considered six observation covariates including year, linear and quadratic forms of ordinal date, occasion number (*j*), minimum temperature (°C), and the bait used (deer or other). All covariates were examined for evidence of collinearity using variance inflation factors; there was no evidence of collinearity between covariates (e.g., Zuur et al. 2009, VIF < 3.00). All continuous covariates were scaled and standardized to have unit variance and a mean of zero. We fit the top models selected for in Twining *et al.* 2024a using AIC-based model selection including linear and quadratic forms of ordinal date but did not include the marten core range covariate which was a proxy for detection probability of martens changing as a function of abundance across their range. Here, using a Royle-Nichols model, we explicitly modelled this relationship between heterogeneity in abundance and detection probability.

Table S8. Table showing the units, description and source of the landscape covariates used in the modelling of the three survey periods from 2016 – 2018 in New York State.

**Landscape covariate**	**Description**	**Source**
Deciduous forest (%)	Proportion of land made up of deciduous trees. (>75% of trees are deciduous, with >20% vegetation cover).	National Landcover Database, 2019 (https://www.mrlc.gov/data/nlcd-2019-land-cover-conus)
Coniferous-mixed forest (%)	Proportion of land made up of the combined class of coniferous and mixed forest classes.	National Landcover Database, 2019 (https://www.mrlc.gov/data/nlcd-2019-land-cover-conus)
All forest (%)	Proportion of land made up of the combined class of deciduous, coniferous, and mixed forest classes.	National Landcover Database, 2019 (https://www.mrlc.gov/data/nlcd-2019-land-cover-conus)
Road density (km)	Mean density of primary and secondary roads per km^2	New York GIS Clearinghouse (https://gis.ny.gov/)
Forest edge (km/km^2)	Edge density of combined class of all forest (coniferous, mixed, and deciduous).	National Landcover Database, 2019 (https://www.mrlc.gov/data/nlcd-2019-land-cover-conus). Calculated using "lsm_c_ed" function in package *landscapemetrics*.
Snow depth (mm)	Averaged daily snow depth across sampling period.	National Operational Hydrologic Remote Sensing Centre, 2004. Snow data assimilation system (SNODAS) products (https://doi.org/10.7265/N5TB14TC).
Minimum temperature (°C)	Averaged daily minimum temperature values across the sampling period.	PRISM Climate data, Oregon State University (http://prism.oregonstate.edu).

Winter severity index (days)	Sum of the days under -17.78°C and the number of days with greater than 38cm of snow depth.	NYSDEC product produced using the National Operational Hydrologic Remote Sensing Centre, 2004. Snow data assimilation system (SNODAS) products (https://doi.org/10.7265/N5TB14TC). PRISM Climate data, Oregon State University (http://prism.oregonstate.edu).
Agriculture (%)	Proportion of land made up of combined class of cultivated crops and pasture.	National Landcover Database, 2019 (https://www.mrlc.gov/data/nlcd-2019-land-cover-conus).

Deer availability covariate

A spatial scale of 2km^2 was used as relevant to the home range size and movement of deer in New York State (Quinn *et al.* 2013). The global model included four observation covariates on detection (occasion number, bait used, both linear and quadratic forms of ordinal date), and five site level covariates on occupancy (forest edge density, road density, agriculture, all forest, and winter severity index, see Table S8 for details). The goodness-of-fit test (Mackenzie & Bailey, 2004) for the white-tailed deer global model provided no evidence of lack of fit ($p = 0.30$), with mild overdispersion ($\hat{c} = 1.14$), and thus QAIC selection with a $\hat{c}$ value of 1.14 was used for the white-tailed deer single species models.

Variation in detection probability of white-tailed deer was primarily a function of linear and quadratic forms of ordinal date ($\alpha_{date} = -1.02 \pm 0.36$; $\alpha_{date^2} = 0.88 \pm 0.34$). Detection varied as a function of the bait used ($\alpha_{deer} = -0.80 \pm 0.21$; $\alpha_{moose} = -0.91 \pm 0.54$; $\alpha_{misc} = -1.70 \pm 0.78$). Detection probability also varied by year with marginal detection probabilities of 0.46 (95% CI = 0.33 – 0.60) in 2016, 0.40 (95% CI = 0.31 – 0.49) in 2017, and 0.57 (95% CI = 0.43 – 0.70) in 2018 (see Table S9).

Marginal occupancy probabilities from the first order models demonstrate that whitetailed deer occupancy was positively associated with road density ($\beta = 0.54 \pm 0.13$) and agriculture ($\beta = 0.45 \pm 0.20$), and negatively associated with winter severity index ($\beta = -0.48 \pm 0.19$, see Table S9). Predicted probability occupancy of white-tailed deer across all sites varied from $\psi = 0.05 – 0.97$.

Table S9. Comparison of detection submodels exploring land cover metrics on occupancy of white-tailed deer (*Odocoileus virginianus*) from a camera trap survey in New York State from January – March 2016 - 2018. Only models with ΔQAIC < 2 are shown. Year is fixed on all models.

**Model**	**-2logL**	**No. parameters**	**QAIC**	**ΔQAIC**	**QAICwt**
**White-tailed deer (*Odocoileus virginianus*)**					
ψ (.), p(Bait, date, date^2)	-740.15	9	1318.50	0.00	0.42
ψ (.), p(Bait, date, date^2, occasion)	-739.16	10	1318.78	0.28	0.36

Table S10. Comparison of final occupancy submodels exploring land cover metrics on occupancy of the white-tailed deer (*Odocoileus virginianus*) from a camera trap survey in New York State from January – March 2016 - 2018. Only models with ΔQuasi Akaike Information Criterion (QAIC) < 2 are shown. Redundant parameters have been removed.

**Model**	**-2logL**	**No. parameters**	**QAIC**	**ΔQAIC**	**QAICwt**
**White-tailed deer (*Odocoileus virginianus*)**					
ψ (Agriculture, road density, winter severity index), p(top)	-708.45	14	1272.90	0.00	0.45
ψ (Agriculture, road density, winter severity index, forest edge), p(top)	-708.41	15	1274.83	1.94	0.17
ψ (Agriculture, road density, winter severity index, all forest), p(top)	-708.44	15	1274.88	1.99	0.17

Visual and Gelman-Rubin diagnostics of chain convergence and posterior predictive checks

Table S11: R-hat values (Gelman & Rubin, 1992) for coyote (*Canis latrans)* – fisher *(Pekania pennanti)* – marten (*Martes americana)* abundance-mediated interaction model using 3 chains of 200,000 iterations with effective thinning of 195,000 per chain.

Parameter	Point estimate	Upper CI
alpha0D[1]	1.02	1.04
alpha0D[2]	1.02	1.05
alpha0D[3]	1.01	1.02
alpha0I[1]	1.00	1.01
alpha0I[2]	1.00	1.01
alpha0I[3]	1.00	1.01
alpha0S[1]	1.00	1.01
alpha0S[2]	1.01	1.03
alpha0S[3]	1.01	1.01
alphaD[1]	1.00	1.01
alphaD[2]	1.00	1.01
alphaD[3]	1.00	1.00
alphaI[1]	1.00	1.00
alphaI[2]	1.00	1.00
alphaI[3]	1.00	1.00
alphaI[4]	1.00	1.01
alphaS[1]	1.00	1.00
alphaS[2]	1.00	1.00
alphaS[3]	1.00	1.00
alphaS[4]	1.01	1.03
beta0D[1]	1.02	1.04
beta0D[2]	1.02	1.05
beta0D[3]	1.01	1.01
beta0I[1]	1.00	1.01
beta0I[2]	1.00	1.01

beta0I[3]	1.00	1.01
beta0S[1]	1.03	1.06
beta0S[2]	1.02	1.05
beta0S[3]	1.00	1.00
betaD[1]	1.00	1.01
betaD[2]	1.00	1.01
betaI[1]	1.00	1.01
betaI[2]	1.00	1.00
betaI[3]	1.00	1.00
betaS[1]	1.00	1.00
betaS[2]	1.00	1.00
betaS[3]	1.00	1.00
gamma0DI	1.01	1.02
gamma0DS	1.00	1.01
gamma0IS	1.00	1.01

Figure S4:

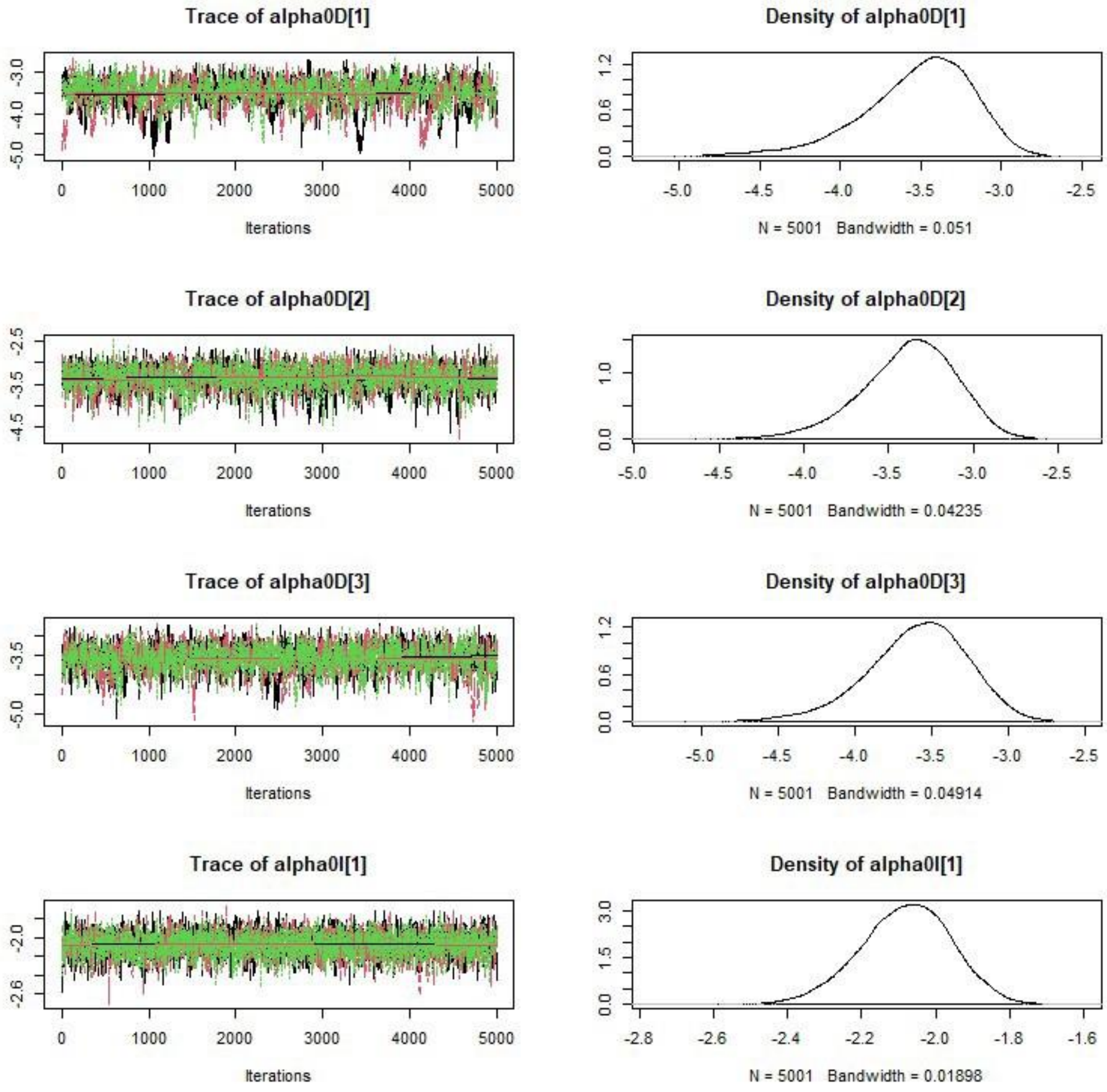
Trace of alpha0D[1]
Iterations
Density of alpha0D[1]
N = 5001 Bandwidth = 0.051
Trace of alpha0D[2]
Iterations
Density of alpha0D[2]
N = 5001 Bandwidth = 0.04235
Trace of alpha0D[3]
Iterations
Density of alpha0D[3]
N = 5001 Bandwidth = 0.04914
Trace of alpha0I[1]
Iterations
Density of alpha0I[1]
N = 5001 Bandwidth = 0.01898

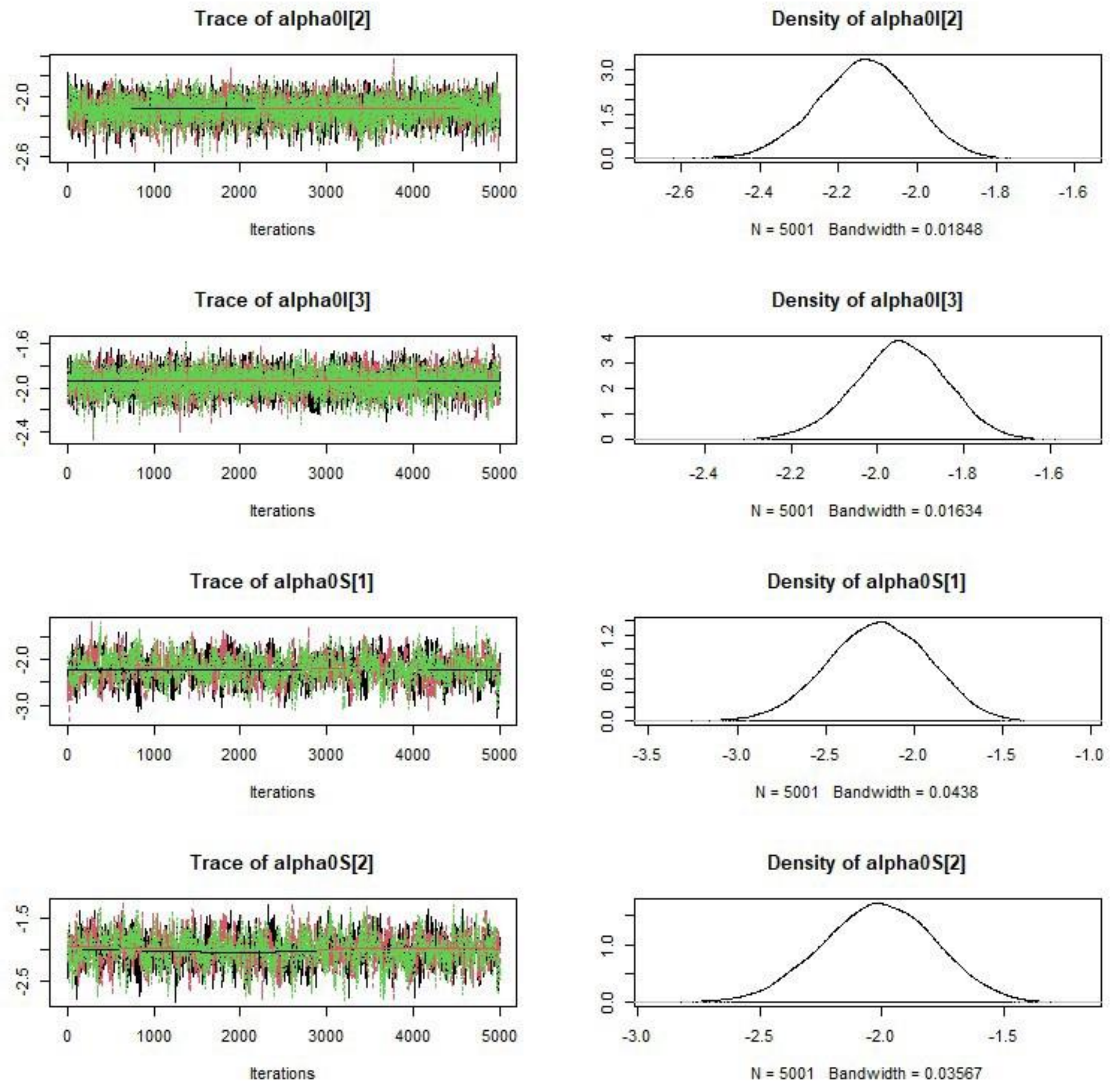
Trace of alpha0I[2]
Iterations
Density of alpha0I[2]
N = 5001 Bandwidth = 0.01848
Trace of alpha0I[3]
Iterations
Density of alpha0I[3]
N = 5001 Bandwidth = 0.01634
Trace of alpha0S[1]
Iterations
Density of alpha0S[1]
N = 5001 Bandwidth = 0.0438
Trace of alpha0S[2]
Iterations
Density of alpha0S[2]
N = 5001 Bandwidth = 0.03567

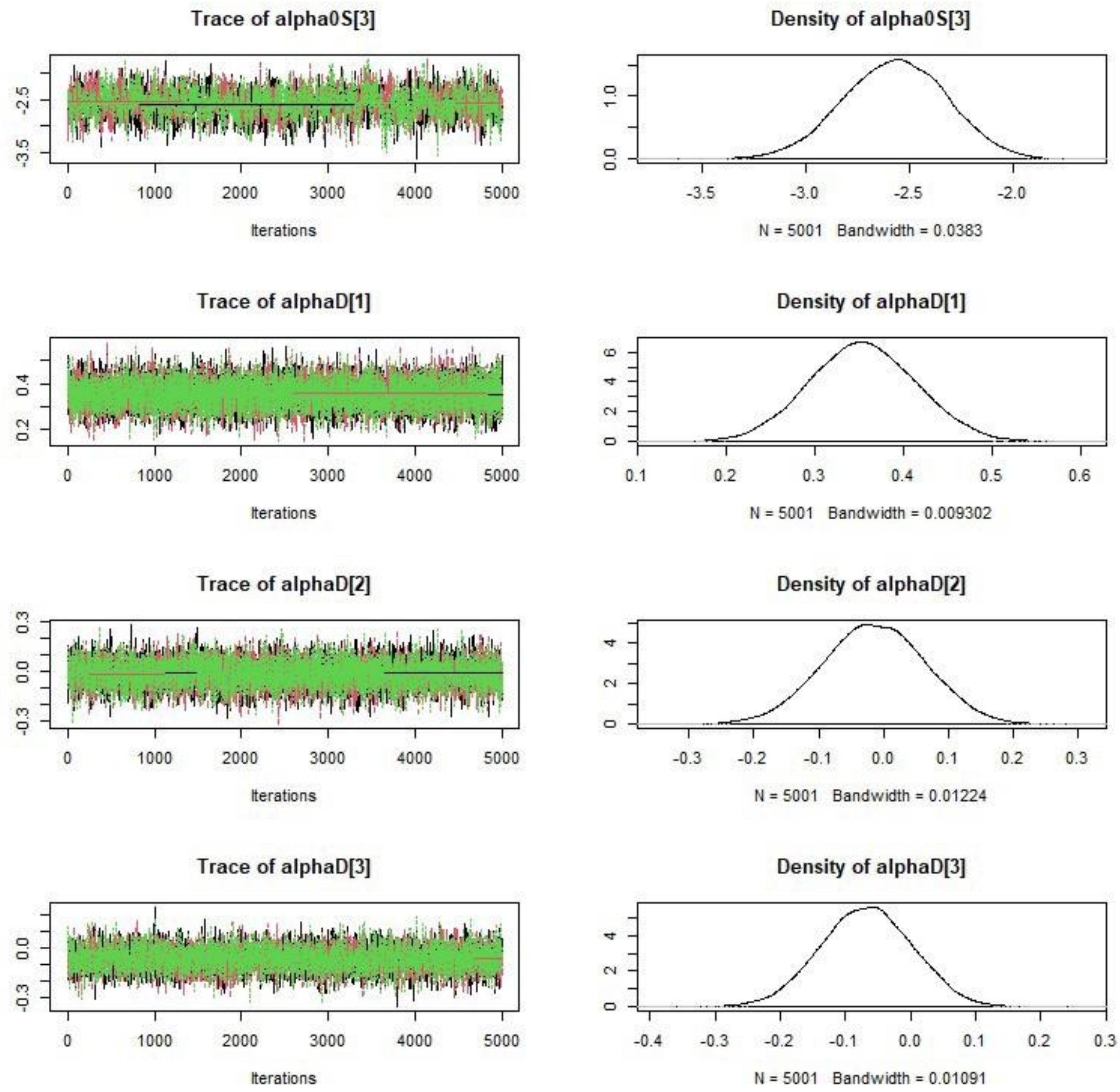
Trace of alpha0S[3]
-3.5
-2.5
0
1000
2000
3000
4000
5000
Iterations
Density of alpha0S[3]
0.0
1.0
-3.5
-3.0
-2.5
-2.0
N = 5001 Bandwidth = 0.0383
Trace of alphaD[1]
0.2
0.4
0
1000
2000
3000
4000
5000
Iterations
Density of alphaD[1]
0
2
4
6
0.1
0.2
0.3
0.4
0.5
0.6
N = 5001 Bandwidth = 0.009302
Trace of alphaD[2]
-0.3
0.0
0.3
0
1000
2000
3000
4000
5000
Iterations
Density of alphaD[2]
0
2
4
-0.3
-0.2
-0.1
0.0
0.1
0.2
0.3
N = 5001 Bandwidth = 0.01224
Trace of alphaD[3]
-0.3
0.0
0
1000
2000
3000
4000
5000
Iterations
Density of alphaD[3]
0
2
4
-0.4
-0.3
-0.2
-0.1
0.0
0.1
0.2
0.3
N = 5001 Bandwidth = 0.01091

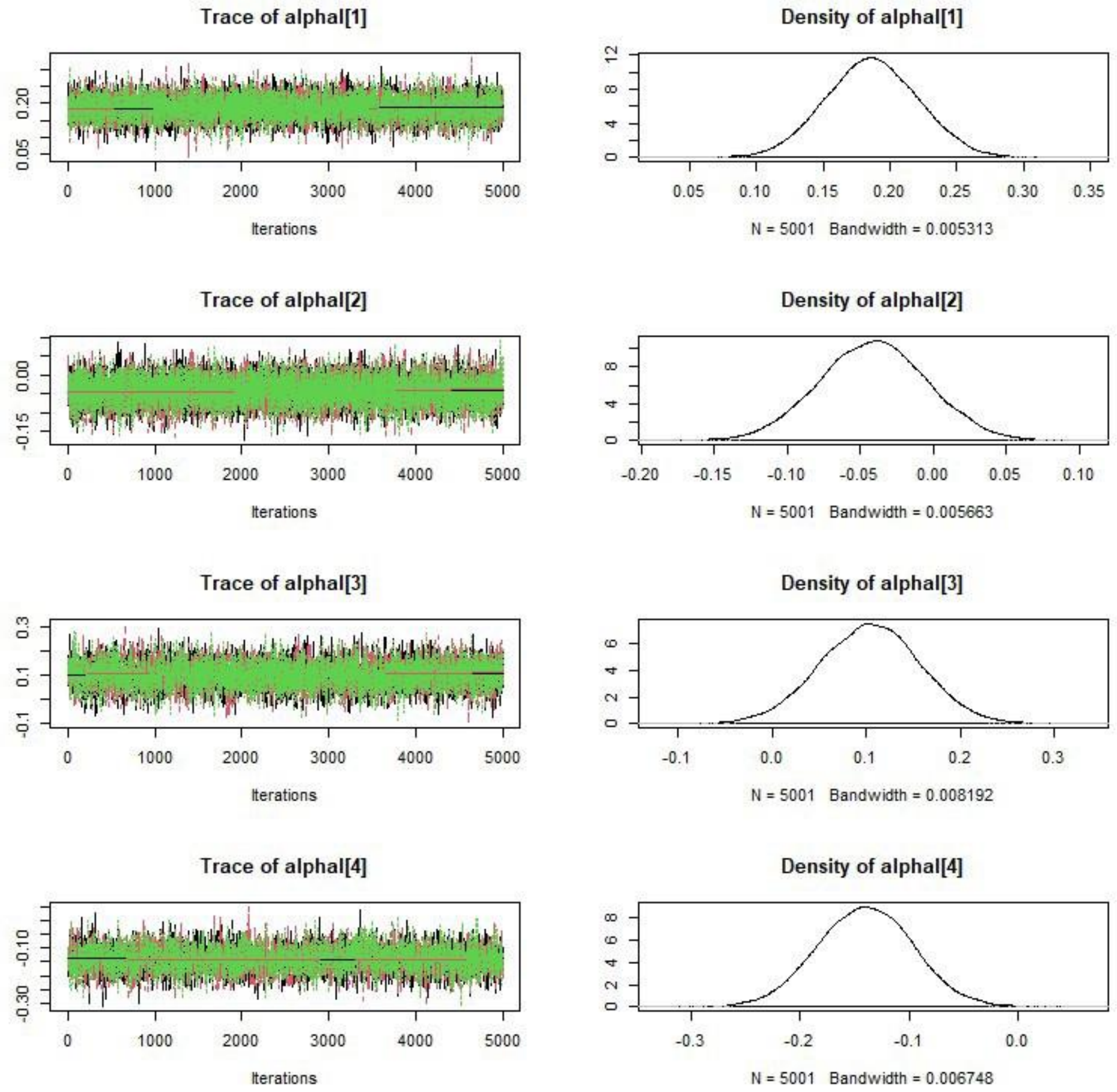
Trace of alphal[1]
Iterations
Density of alphal[1]
N = 5001 Bandwidth = 0.005313
Trace of alphal[2]
Iterations
Density of alphal[2]
N = 5001 Bandwidth = 0.005663
Trace of alphal[3]
Iterations
Density of alphal[3]
N = 5001 Bandwidth = 0.008192
Trace of alphal[4]
Iterations
Density of alphal[4]
N = 5001 Bandwidth = 0.006748

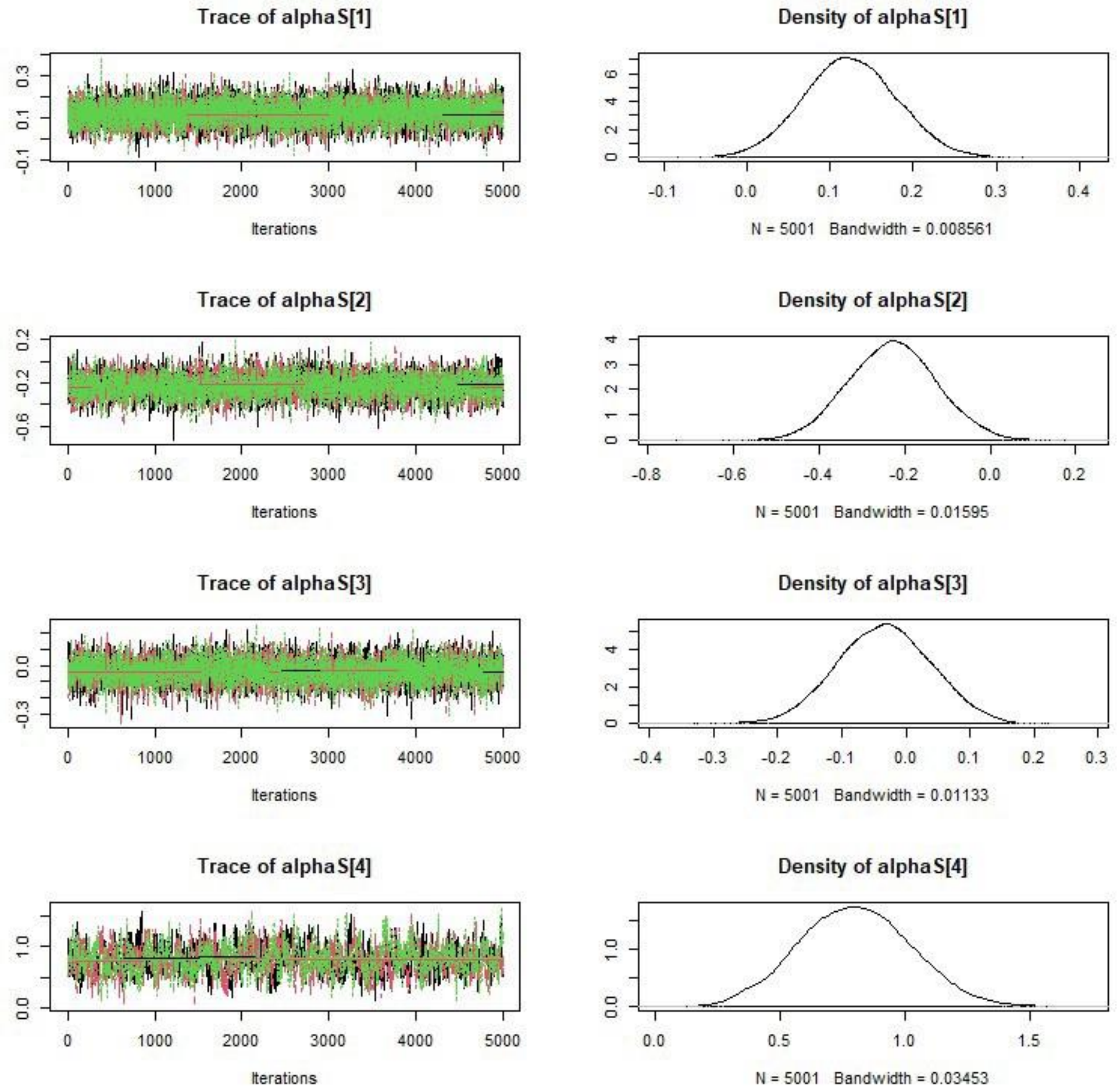
Trace of alphaS[1]
Iterations
Density of alphaS[1]
N = 5001 Bandwidth = 0.008561
Trace of alphaS[2]
Iterations
Density of alphaS[2]
N = 5001 Bandwidth = 0.01595
Trace of alphaS[3]
Iterations
Density of alphaS[3]
N = 5001 Bandwidth = 0.01133
Trace of alphaS[4]
Iterations
Density of alphaS[4]
N = 5001 Bandwidth = 0.03453

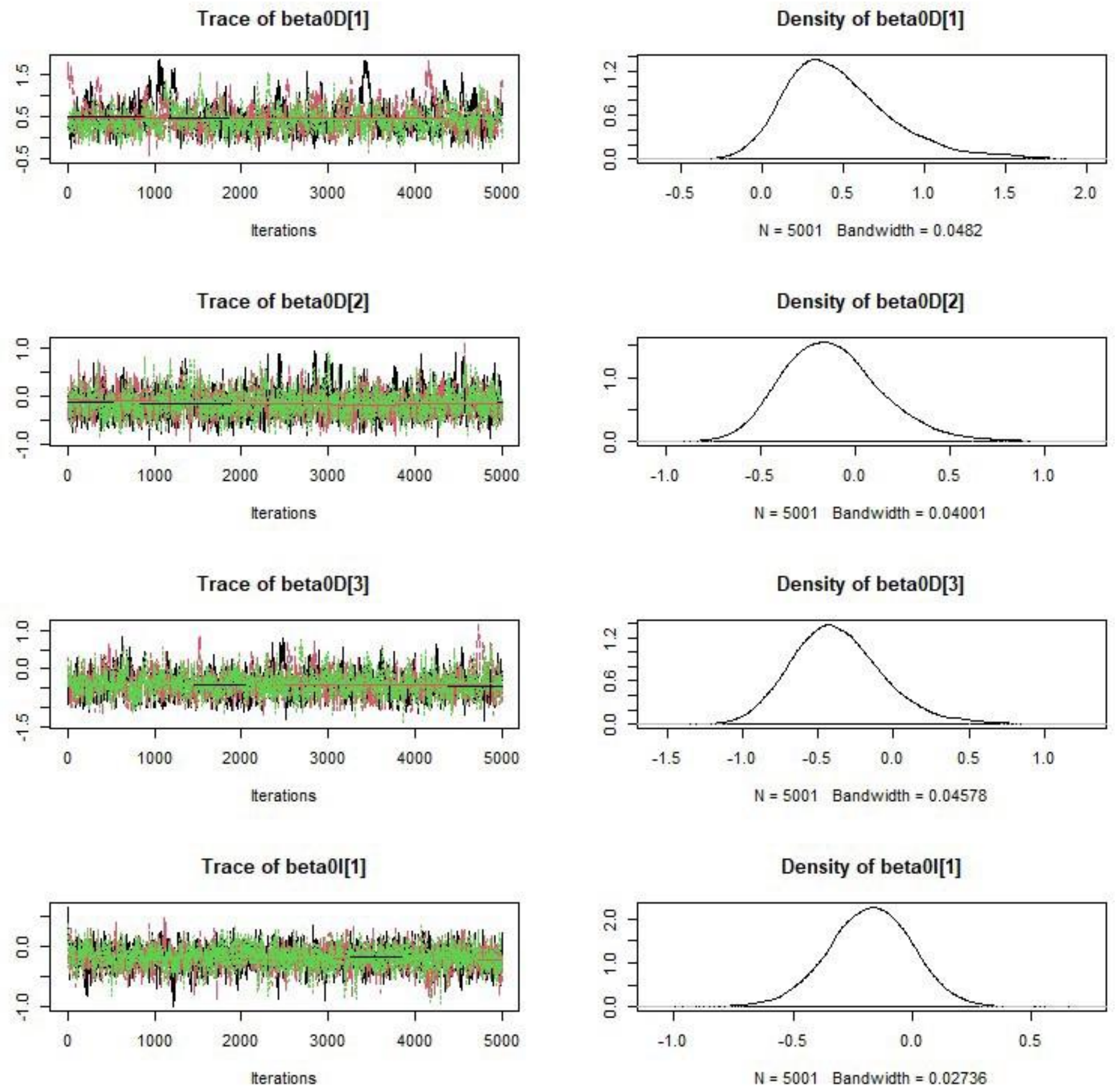
Trace of beta0D[1]
Iterations
Density of beta0D[1]
N = 5001 Bandwidth = 0.0482
Trace of beta0D[2]
Iterations
Density of beta0D[2]
N = 5001 Bandwidth = 0.04001
Trace of beta0D[3]
Iterations
Density of beta0D[3]
N = 5001 Bandwidth = 0.04578
Trace of beta0I[1]
Iterations
Density of beta0I[1]
N = 5001 Bandwidth = 0.02736

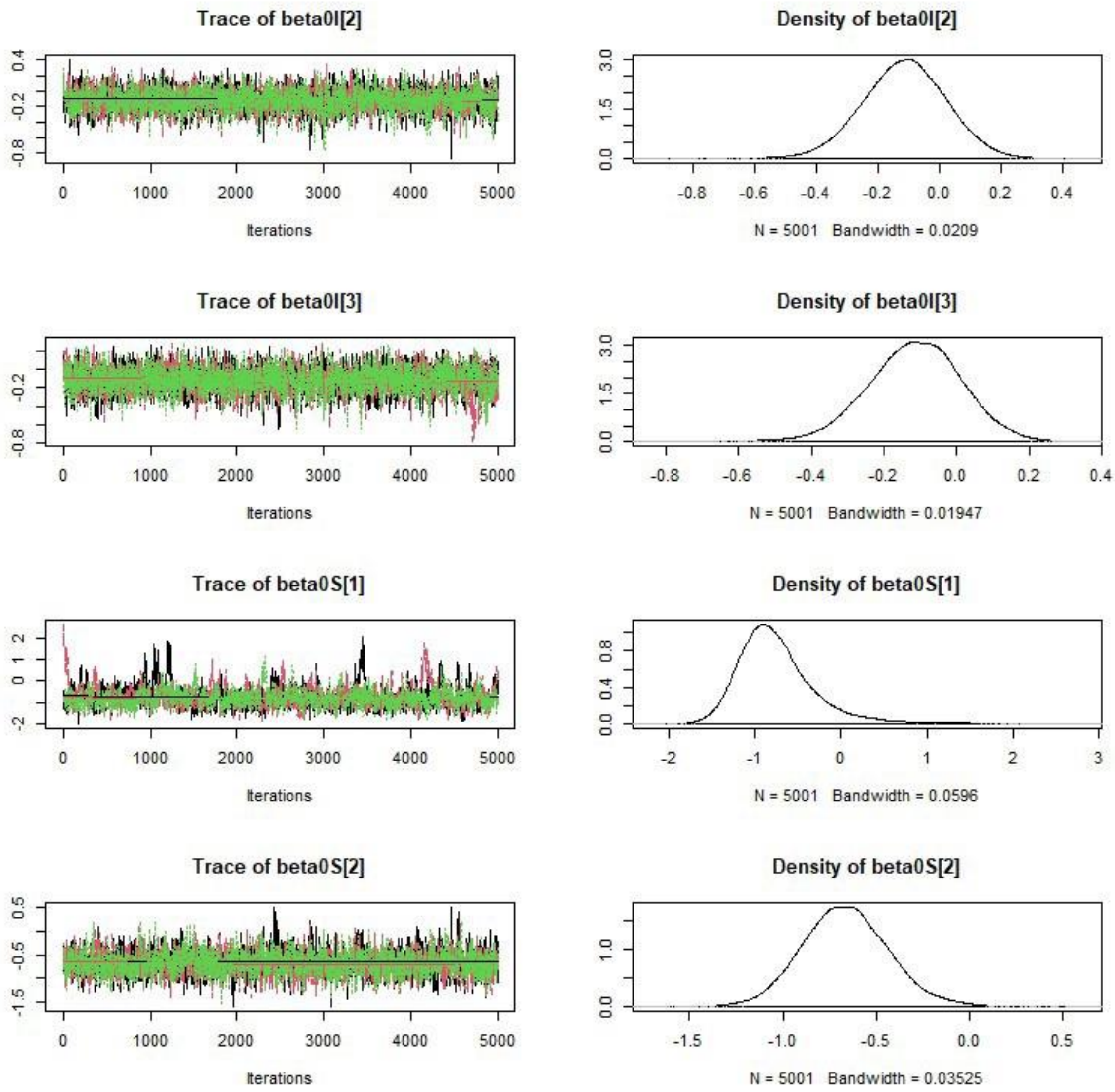
Trace of beta0I[2]
Iterations
Density of beta0I[2]
N = 5001 Bandwidth = 0.0209
Trace of beta0I[3]
Iterations
Density of beta0I[3]
N = 5001 Bandwidth = 0.01947
Trace of beta0S[1]
Iterations
Density of beta0S[1]
N = 5001 Bandwidth = 0.0596
Trace of beta0S[2]
Iterations
Density of beta0S[2]
N = 5001 Bandwidth = 0.03525

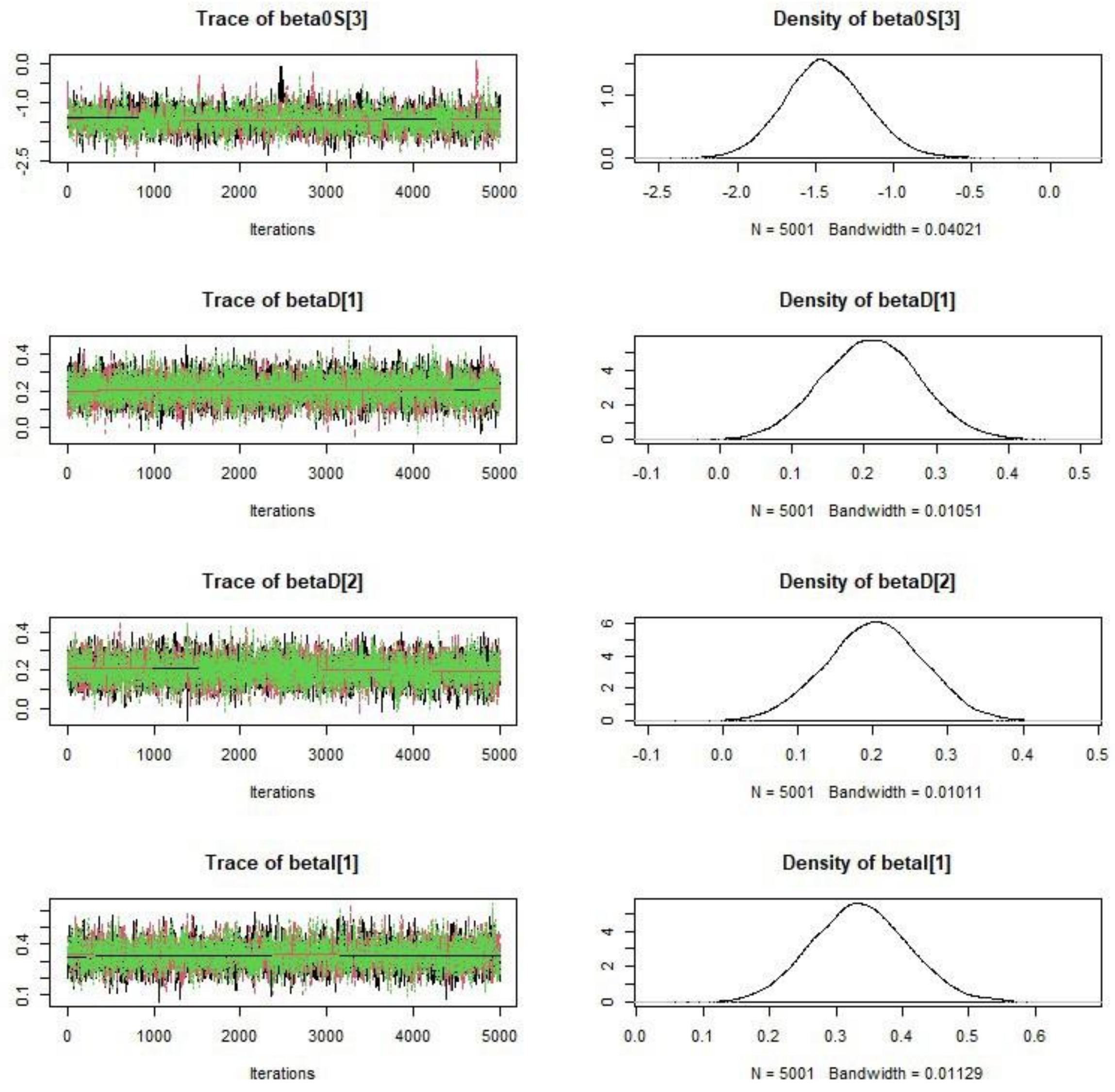

Trace of beta0S[3]
Iterations
Density of beta0S[3]
N = 5001 Bandwidth = 0.04021
Trace of betaD[1]
Iterations
Density of betaD[1]
N = 5001 Bandwidth = 0.01051
Trace of betaD[2]
Iterations
Density of betaD[2]
N = 5001 Bandwidth = 0.01011
Trace of betal[1]
Iterations
Density of betal[1]
N = 5001 Bandwidth = 0.01129

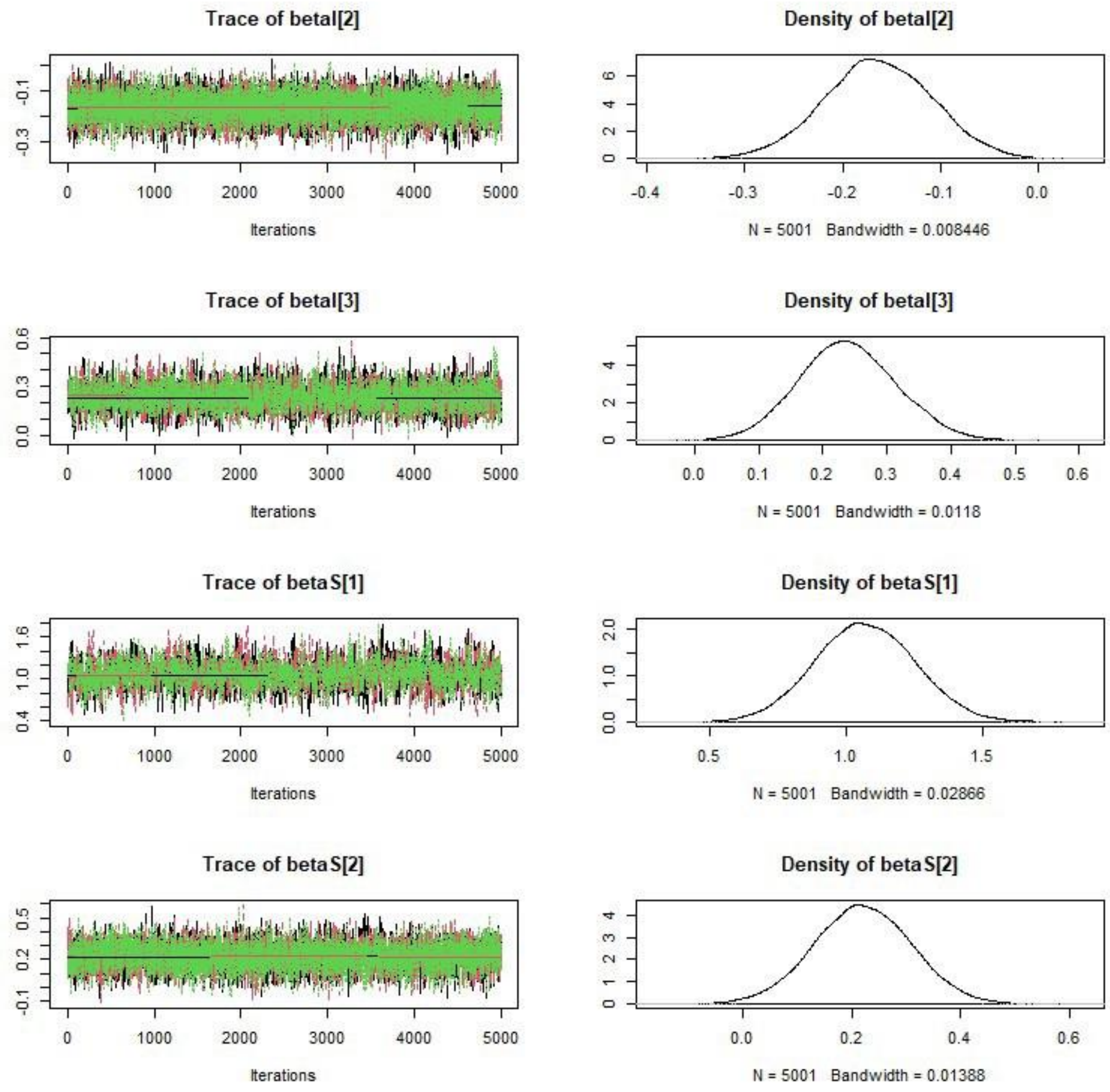

Trace of betaI[2]
Iterations
Density of betaI[2]
N = 5001 Bandwidth = 0.008446
Trace of betaI[3]
Iterations
Density of betaI[3]
N = 5001 Bandwidth = 0.0118
Trace of beta S[1]
Iterations
Density of beta S[1]
N = 5001 Bandwidth = 0.02866
Trace of beta S[2]
Iterations
Density of beta S[2]
N = 5001 Bandwidth = 0.01388

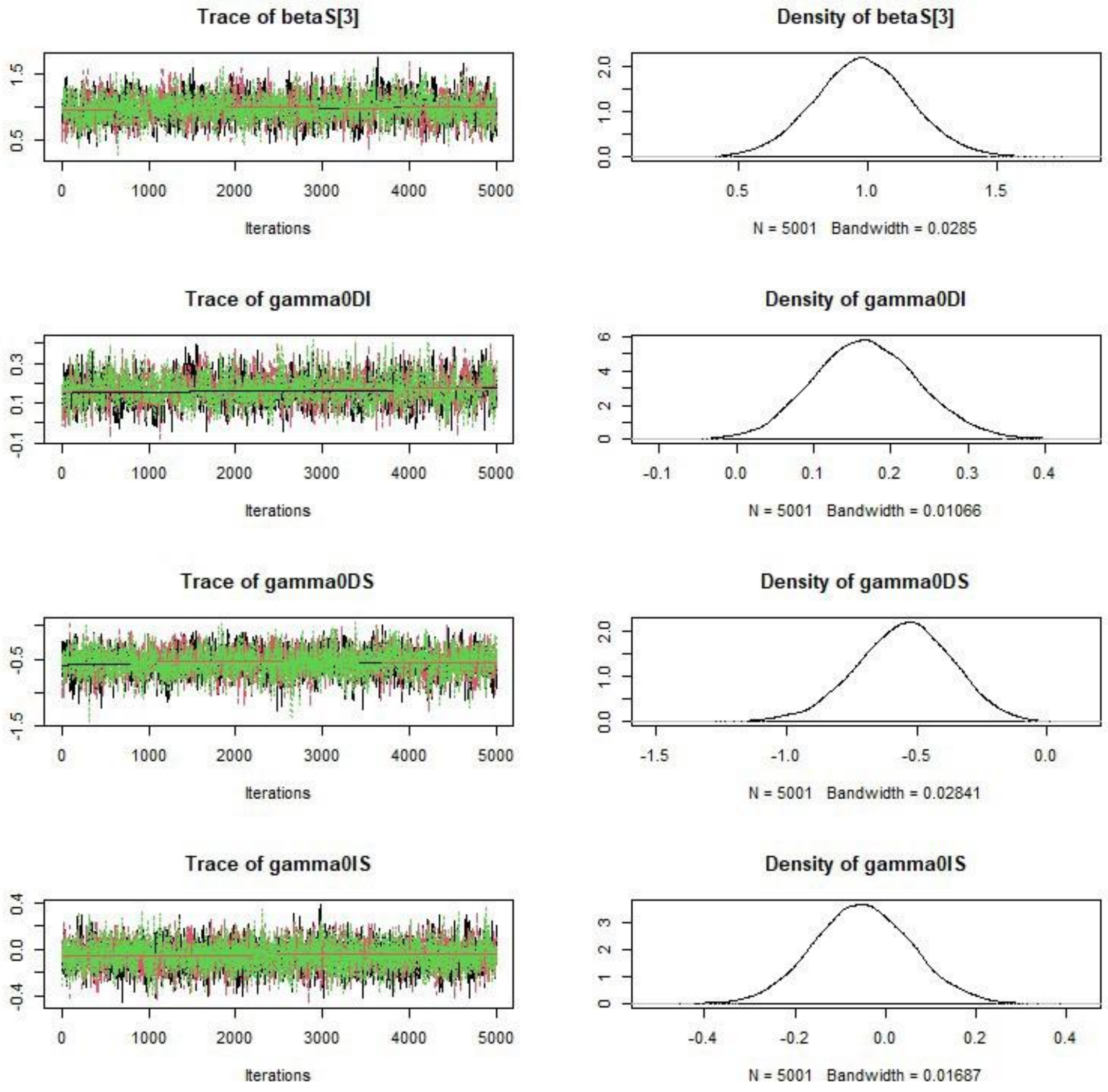

Trace and density plots for the coyote (*Canis latrans)* – fisher *(Pekania pennanti)* – marten (*Martes americana)* occupancy-abundance model using 3 chains of 200,000 iterations with effective thinning of 195,000 per chain.

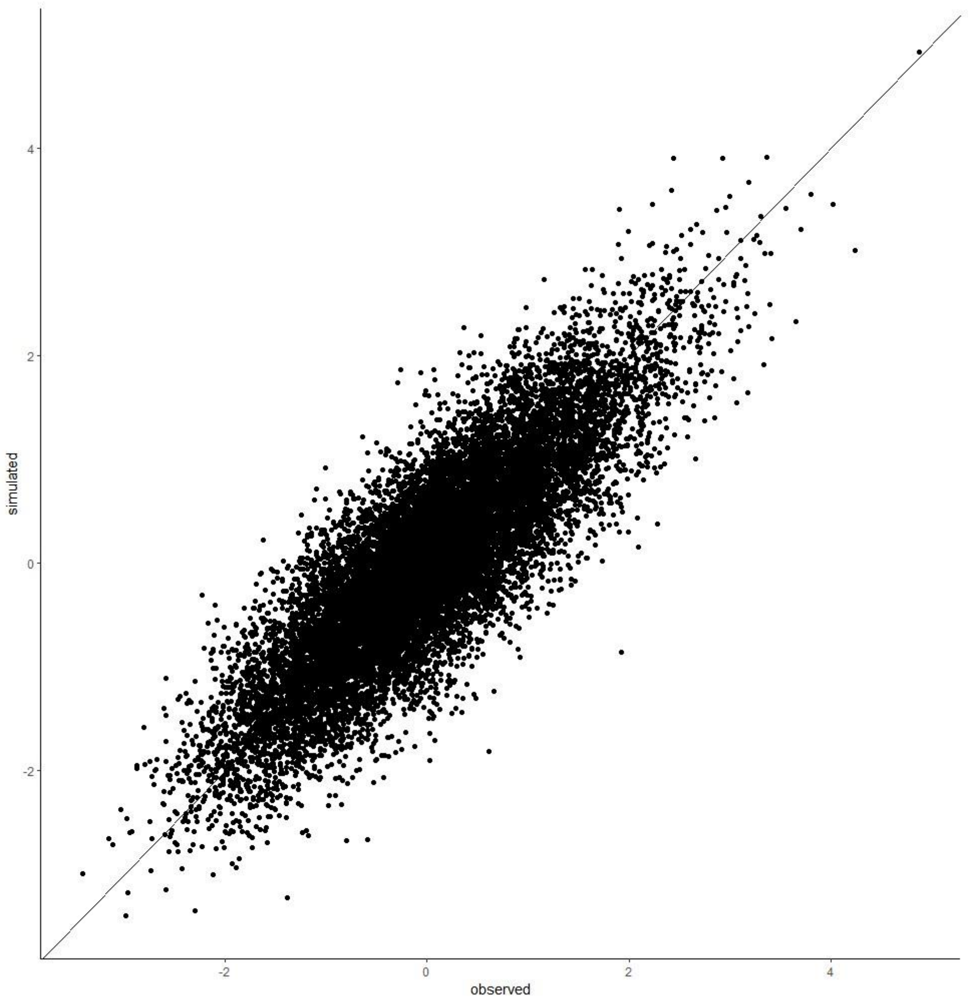

Figure S5: Posterior predictive check for the coyote (*Canis latrans*) submodel using 3 chains of 200,000 iterations with effective thinning of 195,000 per chain.

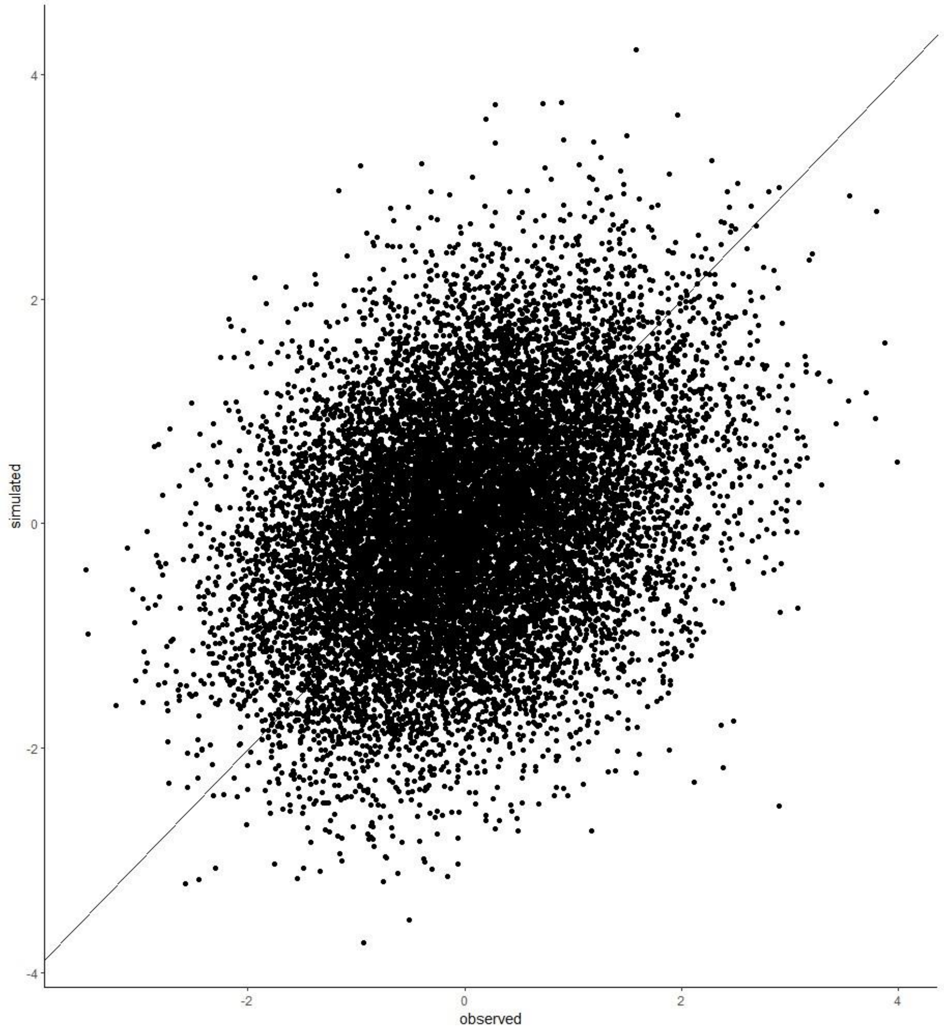

Figure S6: Posterior predictive check for the fisher (*Pekania pennanti*) submodel using 3 chains chains of 200,000 iterations with effective thinning of 195,000 per chain.

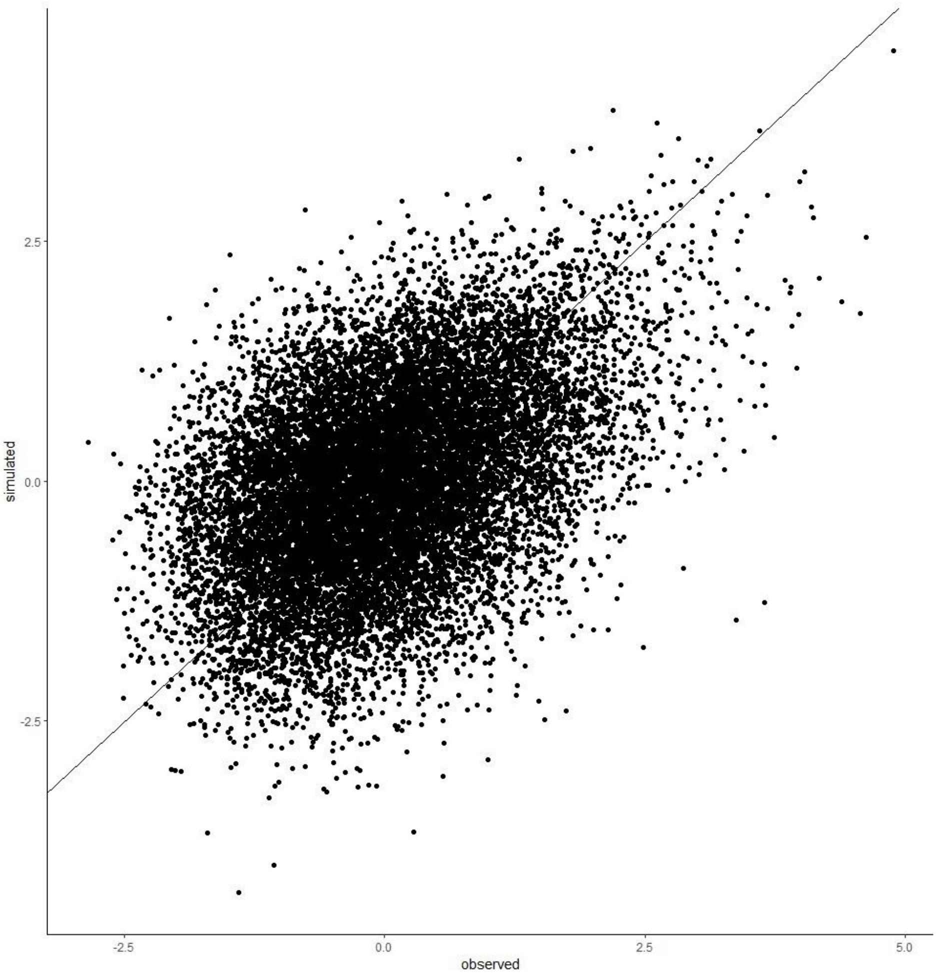

Figure S7: Posterior predictive check for the marten *(Martes americana)* submodel using 3 chains chains of 200,000 iterations with effective thinning of 195,000 per chain.

References

Fuller, A. K., Linden, D. W., Royle, J. A. (2016). Management decision making for fisher populations informed by occupancy modelling. Journal of Wildlife Management, 80: 794–802.

Gelman, A., and D. B. Rubin. (1992). Inference from Iterative Simulation Using Multiple Sequences. Statistical Science, 7(4): 457–72.

Jensen, P. G., Humpries, M. M. (2019). Abiotic conditions mediate intraguild interactions between mammalian carnivores. Journal of Animal Ecology, 88: 1305–1318.

MacKenzie, D.I., Bailey. L.L. (2004). Assessing the fit of site-occupancy models. Journal of Agricultural, Biological, and Environmental Statistics, 9: 300-318.

Siren, A., Sutherland, C., Bernier, C. A., Royar, K. J., Kilborn, J. R., Callahan, C., Cliche, R. M., Prout, L. S. and Morelli, T. L. (2021). Abiotic stress and biotic factors mediate range dynamics on opposing edges. Journal of Biogeography, 48: 1758–1772.

Twining *et al.* (2024a). Intraguild interactions and abiotic conditions mediate occupancy of mammalian carnivores: co-occurrence of coyotes-fishers-martens. *Oikos*, 2024(6): e10577.

Quinn, A.C.D., Williams, D.M., Porter, W.F. (2013). Landscape structure influences space use by white-tailed deer. Journal of Mammalogy, 94: 398-407.

Zuur, A. F., Leno, E. N., Walker, N. J., Saveliev, A. A., Smith, G. M. 2009. Mixed effects models and extensions in ecology with R. Springer.
